# Impact of *Sporisorium scitamineum* infection on the qualitative traits of commercial cultivars and advanced lines of sugarcane

**DOI:** 10.1371/journal.pone.0268781

**Published:** 2022-05-23

**Authors:** Muhammad Aslam Rajput, Rehana Naz Syed, Fahad Nazir Khoso, Jamal-U-Din Hajano, Nasir Ahmed Rajput, Muhammad Ali Khanzada, Abdul Mubeen Lodhi

**Affiliations:** 1 Faculty of Crop Protection, Sindh Agriculture University, Tandojam, Pakistan; 2 National Sugar and Tropical Horticulture Research Institute, PARC, Thatta, Pakistan; 3 Department of Plant Pathology, University of Agriculture, Faisalabad, Pakistan; Ghazi University, PAKISTAN

## Abstract

Whip smut disease of sugarcane, caused by *Sporisorium scitamineum*, is considered one of the main constraints in the successful cultivation of sugarcane. The pathogen infection can decrease the quantity and quality of the produce. Cultivation of resistant varieties is the most feasible strategy to combat the harms of this devastating disease. Development of varieties having disease-resistance together with improved important traits such as brix, pol, purity, CSS, and low fiber contents are desirable. Therefore, we documented the variances in quality traits of 104 sugarcane cultivars under disease pressure in split-plot design with 6 replications. The split ANOVA revealed a highly significant impact (p<0.0001) between treatments (inoculated and uninoculated), within cultivars as well as interaction ‘Cultivars x Treatments’ effect on brix, pol, fiber, purity, and CSS contents. In inoculated plots, the infection of *S*. *scitamineum* brought a highly significant reduction (t>4.032) in brix, pol, purity, and CSS of more than 40% of the cultivars used, as compared to the uninoculated ones. On the other hand, the smut infection caused a highly significant (t>4.032) increase in fiber percentage of 41 cultivars. We found significant positive correlations between smut rating and reduction of brix, pol, purity, and CSS contents. The cultivars that were caught with greater disease severity, compromised a higher reduction of their useful contents. Similarly, a significant positive correlation was found between increased fiber percent and smut rating. Remarkably, cultivars that showed immune reactions to whip smut disease were not statistically different from uninoculated ones in brix, pol, purity, CSS, and fiber contents. Variable effects of whip smut infection to quality parameters of different cultivars depict the importance of further improvement through breeding programs.

## Introduction

Sugarcane (*Saccharum officinarum* L.) is classified in the grass family Poaceae along with other economically important plant species including wheat, rice, barley, oats, rye, etc. It is the world’s major sugar crop, widely cultivated in subtropical to tropical regions at 30° S latitude to 30° N latitude [1]. A mean temperature of 28 to 32°C is best suitable for its growth, temperature above 50°C or below 20°C can detain its growth. Relative humidity of 70 to 85 percent during growth and 55 to 75 percent during the ripening phase is ideal. Relative humidity less than 50 percent during the growing season is not suitable for sugarcane cultivation [2]. It is cultivated on about 26.47 million hectares, with a worldwide yield of 1869.72 million tonnes [3] and its cultivation has been persistently expanding [4]. Americas and Asia’s regions are the major contributors, shares 51.85 and 40.61% in area, and 53.98 and 39.17% in production, respectively. Sugarcane is the main source of sucrose, contributed about 80% of the world’s total sugar production [3]. Sugarcane by-products also have a significant role as alcohol in medicines, ethanol for biofuel production, molasses for bakery items and animal feed, bagasse for paper and press mud for organic matter engendering [5–9]. About 40% of the world’s total ethanol fuel is obtained from sugarcane. In Pakistan, sugarcane ranked as the second major cash crop after cotton, which has been contributed 0.7% to gross domestic production [10]. During 2020–21, sugarcane was grown on about 1.165 million hectares with cane production of 81 million tonnes and sugar production of 7.5 million tonnes [11].

The sugarcane crop requires 12–14 months for maturity and harvesting, therefore being a long duration crop, it suffers from many biotic and abiotic factors that affect its productivity. The biotic factors (insect pests and diseases) have the potential to decrease production by 19 and 20%, respectively [12–14]. More than a hundred diseases of sugarcane have been reported from different parts of the world [13, 15]. Of all the sugarcane diseases, fungal diseases are the most prevalent pest of sugarcane crop and gaining internationally more attention [16]. Among various disease-causing organisms, *Sporisorium scitamineum* (Syd.) M. Piepenbr., M. Stoll & Oberw (syn. *Ustilago scitaminea*) is an obligate parasite that causes destructive whip smut disease in almost all the cane growing regions of the world [17, 18]. After the first appearance in the Natal region of South Africa during 1877, the disease has been spread to other cane growing regions of the world except for Papua New Guinea and Fiji [17, 19, 20]. This smut disease is characterized by the formation of a black curved shoot instead of a cane. Therefore, in most cases, the successful infection has resulted in the total loss of a millable cane. The whip smut is known to affect both qualitative and quantitative components, which ultimately caused substantial economical losses [21–23]. Losses can range from 30% to total crop failure, and the disease even leads to variety elimination due to susceptibility to this fungus [24]. Susceptible cultivars show significant losses due to poor management practices, secondary infection, and intensive cultivation. Variable losses are reported mostly due to the different cultivars and climatic conditions such as 10–30% yield and 3–20% sugar losses [25], 68–80% yield and 32% in juice quality [26], 62% in yield [27], 40–90% in sugar [28]. In extreme cases, complete crop failure may happen [29]. Besides the direct yield loss, the whip smut can cause a significant reduction in sucrose recovery, purity, and other juice quality indicators [30–32]. The most reliable, cost-effective, and eco-friendly disease control can only be achieved through resistant varieties [33]. Therefore, screening sugarcane germplasm for smut resistance and other desirable characteristics is an ongoing process. The present study focuses on the impact of *S*. *scitamineum* on the qualitative parameters of a wide range of sugarcane cultivars grown both in infested and pathogen-free conditions.

## Materials and methods

### Experimental site and source of cultivars

The field experiment was carried out in the field area of Agriculture Research Institute, Tandojam located in the southeast province (Sindh) of Pakistan (25^0^ 25.19 N; 68^0^ 32.07 E). The experimental site has a long history of wheat-cotton rotation, which means no cane cultivation in past. This study was undertaken for two consecutive seasons (2014–15 and 2015–16).

Indigenous and exotic sugarcane germplasm were collected from different sources such as National Sugar & Tropical Horticulture Research Institute (NSTHRI) Thatta, Nuclear Institute of Agriculture (NIA) Tandojam and Agriculture Research Institute (ARI) Tandojam (Table 1). Planting material was obtained from a one-year old crop, which was completely free from smut disease. To minimize the chances of contamination of planting materials with soil-borne propagules of smut pathogen, before treatment and sowing all planting materials were subjected to hot water treatment (52°C for 30 minutes) to eliminate any setts borne pathogen inoculum [34].

**Table 1:** List of cultivars grown under field conditions for two growing seasons (2014–15 and 2015–16).

S. No	Varieties/ Cultivars	Source	S. No	Varieties/ Cultivars	Source
1	HoTh-409	NSTHRI, Thatta	53	NIA-2004	
2	Th-725		54	Chandka	QAARI, Larkana
3	BPTh-807		55	Larkana-2001	
4	HoTh-550		56	S-2006-SP-30	SRI, AARI, Faisalabad
5	HoTh-516		57	S-2006-SP-18	
6	Th-702		58	CPF-229	
7	HoTh-424		59	CP-85-SP-571	
8	HoTh-316		60	S-2003-US-633	
9	BPTh-804		61	S-2003-US-160	
10	HoTh-408		62	HSF-240	
11	HoTh-513		63	S 2003-US-704	
12	HoTh-517		64	CP-70-SP-1215	
13	HoTh-419		65	S-2002-SFSD-1307	
14	HoTh-518		66	CPF-134	
15	HoTh-432		67	CO-208	SBRI, Coimbatore (India)
16	HoTh-401		68	NCO-310	
17	HoTh-326		69	CO-639	
18	Th-720		70	CO-620	
19	HoTh-544		71	CO-413	
20	Th-704		72	CO-1148	
21	HoTh-127		73	H-86-NSG-311	SSRI, Jhang
22	HoTh-518		74	SPSG-3481	
23	HoTh-612		75	S-2003-CPSG-704	
24	HoTh-344		76	S-2002-HSG-200	
25	HoTh-610		77	CPSG-244-S-2083	
26	HoTh-4140		78	S-2003-QSSG-776	
27	Th-10		79	CSSG-2402	
28	Q-88	Sugarcane Section, ARI Tandojam	80	QSG-1741	
29	AP-98-156/01		81	CSSG-1741	
30	AP-98-156/02		82	S-2003-HOSG-701	
31	AP-98-156/03		83	S-2003-HOSG-1626	
32	AP-98-156/04		84	COJ-84	
33	AP-98-156/05		85	S-2003-CPSG-193	
34	AP-98-156/06		86	NSG-60	
35	AP-04-59/01		87	CSSG-2476	
36	AP-04-59/02		88	S-2003-HOSG-679	
37	AP-04-68/01		89	COJ-81	
38	AP-98-156/07		90	SPSG-26	
39	AP-98-156/08		91	YT-236	GARI,China
40	AP-98-103/01		92	Roc-16	
41	AP-97-69/ 01		93	CPS-1827	SCRI, Mardan
42	AP-97-56/02		94	CP-70-530	
43	AP-97-56/03		95	CP-59-1059	
44	AP-04-46/02		96	CP-29-120	
45	AP-04-46/03		97	CP-82-2083	
46	AP-04-59/03		98	CP-52-28	
47	AP-04-68/02		99	CP-69-1059	
48	AP-04-68/03		100	CP-75-1353	
49	BP-TJ-15/01		101	Tritan	
50	BP-TJ-651/18		102	CB-2919	Campos, Brazil
51	BP-TJ-651/20		103	B-43405	Barbados
52	NIA-98	NIA, Tandojam	104	B-46364	

NSTHRI = National Sugar & Tropical Horticulture crops Research Institute, ARI = Agricultural Research Institute, NIA = Nuclear Institute of Agriculture, QAARI = Quaid-Awam Agriculture Research Institute, SRI, AARI = Sugarcane Research Iinstitute, Ayub Agriculture Research Institute, SSRI = Shakarganj Sugar Research Institute, SCRI = Sugar Crops Research Institute, SBRI = Sugarcane Breeding Research Institute, GARI = Guangzhou Agricultural Research Institute.

### Land preparation and experimental design

*S*. *scitamineum* is a soil-borne pathogen, therefore, the experiment was carried out in the soil which had no previous history of sugarcane plantation and arranged in a split-plot design. This was done to consider that the soil was free from *S*. *scitamineum* and to avoid cross-contamination. The soil texture and chemical properties are best suited for cane cultivation. Prior to sowing, all standard agronomic practices were followed to prepare the land. A total of 104 cultivars of different origins were grown with 6 replications in which disease treatments (inoculated & uninoculated) served as whole plots (Factor A) and cultivars as sub-plots (Factor B), randomized within each block. Each subplot consisted of a single row of 5- meter length, with a row to row distance of one meter. About 13 (3-budded) setts of each cultivar were sown in the ridges so that the standard seed rate of 80000 buds per hectare was achieved [35, 36]. The length of each block was measured from the first row to the last row of the plot, which made the plot size 5×104×6 = 3120 square meters excluding paths, etc.

### Pathogen inoculation

Smut whips were collected from infected sugarcane fields of all the cane growing regions of the Sindh province to ensure the presence of all *S*. *scitamineum* present in a study area. The teliospores were gently scraped from shade-dried whips and stored in the refrigerator for future use. At the time of inoculation, their viability was confirmed on water agar plates to be 90% [37]. For inoculation, spore suspension was prepared in large sterilized stainless steel containers, filled with 50 liters of distilled sterilized water, amended with teliospores, and a few drops of Tween 20 to homogenize the suspension. The inoculum density was set to 5×10^6^ spores ml^-1^ with the help of a hemocytometer [38]. The three budded setts of each cultivar were dipped into spore suspension for 30 minutes and then kept in polythene bags for overnight to provide favorable conditions for spore penetration [17, 39, 40]. The sets dipped in distilled-sterilized water having no pathogen inoculum, served as control (un-inoculated).

### Disease scoring

Smut clumps and whips that appeared were counted and roughed out after each observation and destroyed to avoid secondary infestation. Disease incidence was computed by using the following formula:

$$\text{Disease}\;\text{incidence}\left(\%\right)=\left(\text{Number}\;\text{of}\;\text{infected}\;\text{stools}/\text{Total}\;\text{number}\;\text{of}\;\text{stools}\right)\times 100$$

The resulting disease incidence then converted into 0–9 disease rating scale [41], where 0: no disease, 1: 0.1–2.5%, 2: 2.6–5.5%, 3: 5.6–7.5%, 4: 7.6–12.5%, 5: 12.6–15.5%, 6: 15.6–18.0%, 7: 18.1–22.5%, 8: 22.6–25.5% and 9: 25.6–100%.

### Qualitative observation

The trial was harvested 14 months after planting, at the time when sucrose accumulation in the cultivars was optimal. The qualitative parameters such as brix, pol, purity, fiber, and CCS were calculated by taking five stalks per furrow.

The canes were crushed with the help of a Cutter grinder (Fabricator) (Model No. SCF-L4, Smith Crafts Fabricator, Gujranwala, Pakistan) to obtain at least 2 kg of crushed material for quality analysis. Five hundred grams of crushed canes were pressed in the hydraulic press (Model No. SCF-HP-06, Smith Crafts Fabricator, Gujranwala, Pakistan); the yielded sugar juice was collected in a 500 ml glass beaker and fiber cake was removed to calculate fiber contents.

#### Fiber

The press cape residues were weighed to find out the bagasse percent of cane and a sub-sample of the same material was used to calculate the moisture percentage in bagasse. 100 grams of the material from the press cape residue was utilized and placed in a Petri dish. The Petri dish plate was then deposited in an oven and dried at a temperature of 110 to 120^0^ F for about 90 to 120 minutes until the bagasse weight become constant. The loss in bagasse weight after drying provides the percentage of moisture in bagasse and fiber percent was determined as suggested by Chen and Chou [42]:

#### Brix

A cane juice sample was collected in a 500 ml beaker. A drop of juice was placed on the prism of the Refractometer (PR-101, ATAGO Co. Ltd, Japan) with the help of a pipette to measure the brix percentage of the sample.

#### Purity

The purity of cane juice is measured for an idea about the maturity of the cane sample or deterioration by the following equation [43]:

$$\text{Purity}\left(\%\right)\text{of}\;\text{juice}=\left(\text{Pol}\;\%\;\text{of}\;\text{juice}/\text{Brix}\;\%\;\text{of}\;\text{juice}\right)\times 100$$

#### Commercial Cane Sugar (CCS)

CCS of the samples determined by using the following formula [44]:

$$\text{CCS}\left(\%\right)=3\text{P}/2[\left(1-\left(\text{F}+5\right)/100\right]-[\text{B}/2\left(1-\text{F}+3\right)/100]$$

Where P stands for the pol percentage of the first-expressed juice, B is the brix percentage of the first-expressed juice and F is the fiber percentage in the cane.

### Data analysis

Statistical parameters such as mean, standard deviation, analysis of variance, LSD multiple comparison tests, paired t-test, and regression equations were calculated by using the Statistix-8.1 analytical software.

## Results

### Effects of *S*. *scitamineum* on brix

Analysis of variance showed that for brix, there was a highly significant difference between treatments (DF = 1, F = 443.98, P = 0.0000), among cultivars (DF = 103, F = 108.07, P = 0.0000) and the interactive response of treatments and cultivars (DF = 103, F = 11.22, P = 0.0000) (Table 2). In aggregate, significantly higher mean brix was recorded in un-inoculated plots (21.81%) as compared to the pathogen-inoculated treatments (20.69%) (Table 3). Brix % of all 104 cultivars in-field screening trial in both treatments, as well as calculated reduction and T-values are given in S1 Table. In inoculated plots, maximum mean brix was recorded in sugarcane cultivars BPTh-807 (23.31±0.08%) followed by HoTh-318 (23.23±0.07%), HoTh-516 (23.20±0.08%) and BP-TJ-651/20 (22.96±0.23%); while minimum brix was noted in CP-52-28 (17.12±0.19%) followed by Co-639 (17.24±0.33%), Co-413 (17.35±0.23%), CoJ-84 (17.54±0.34%), AP-98-156/08 (17.60±0.24%) and CP-69-1059 (17.71±0.32%). There was no significant difference between brix percent of inoculated and un-inoculated cultivars, which were immune (S1 Table).

**Table 2:** Analysis of variance of cultivars and disease treatments for brix contents.

Source of variation	DF	SS	MS	F-Ratio
A (Treatments)	1	389.76	389.762	443.98*
Blocks (Replicates)	5	3.77	0.754	
Error A (Blocks*Treatments)	5	4.39	0.878	
B (Cultivars)	103	2628.56	25.520	108.07*
A*B (Treatments*Cultivars)	103	272.79	2.648	11.22*
Error B (Blocks*Treatments*Cultivars)	1030	243.22	0.236	
Total	1247	3542.49		

* p<0.0001.

**Table 3:** The main effects of disease treatments on average qualitative parameters of sugarcane cultivars.

Parameters	Treatments		LSD
	Inoculated	Un-inoculated	
Brix	20.690^b^	21.808^a^	0.1364
Pol	17.152^b^	18.429^a^	0.1428
Fiber	13.127^b^	13.726^a^	0.0728
Purity	82.784^b^	84.402^a^	0.2735
CSS	12.120^b^	12.989^a^	0.0849

Note: The data is compared horizontally. The data with different letter are significantly different from one another.

**Fig 1:**
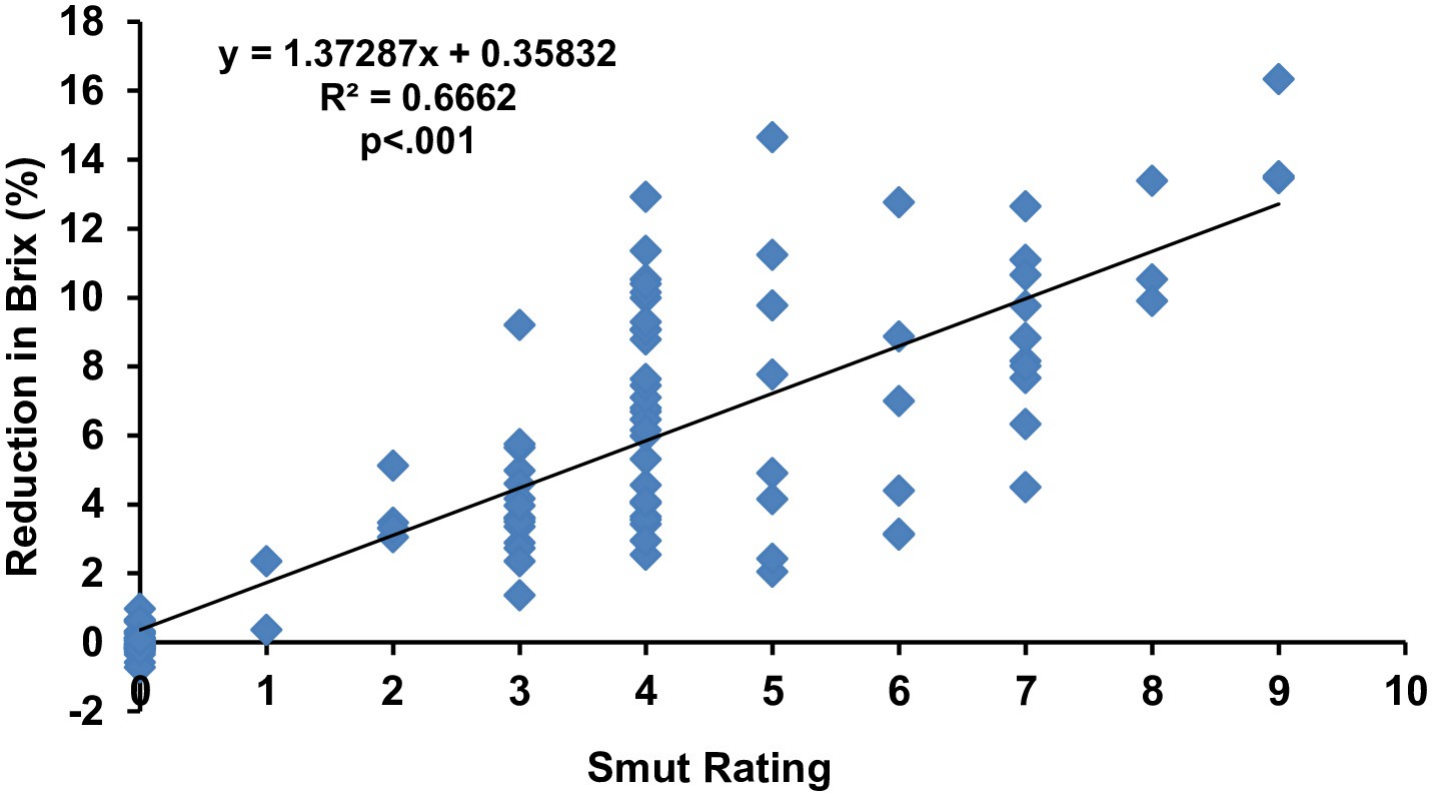
Regression analysis of brix percentage with whip smut disease rating. Note: The smut rating is not a linear scale. It’s based on the transformation explained in methods from linear incidence value to the rating. Accordingly, the resulting disease incidences were converted into 0–9 disease rating scale [41], where 0: no disease, 1: 0.1–2.5%, 2: 2.6–5.5%, 3: 5.6–7.5%, 4: 7.6–12.5%, 5: 12.6–15.5%, 6: 15.6–18.0%, 7: 18.1–22.5%, 8: 22.6–25.5% and 9: 25.6–100%.

The least-square linear regression analysis (Fig 1) showed that the smut rating and the reduction percentage of brix were moderately related to each other (R^2^ = 0.6662) and disease rating is influencing 67% reduction of brix of sugar cane. The statistical analysis further indicated that there is a significant (p<.001) effect of disease incidence on brix. Moreover, according to the regression equation (y = 1.3728x + 0.3583), it can be predicted that with the increase of single rating in the disease, 1.3728 percent of variation can be observed in brix percentage.

### Effects of *S*. *scitamineum* on pol

In terms of pol percentage, analysis of variance revealed a highly significant difference between inoculated (17.15%) and un-inoculated (18.43%) treatments. The cultivars main effect (DF = 103, F = 141.15, P = 0.000), pathogen treatment’s main effect (DF = 1, F = 528.57, P = 0.000) and cultivars x treatment’s effect (DF = 103, F = 16.94, P = 0.000) are highly significant (Tables 3 and 4). Pol of all 104 cultivars in-field screening trial in both treatments as well as resulting reduction and t value are given in S2 Table. In inoculated plots, maximum mean pol was noted in BPTh-807 (20.04±0.03%) followed by HoTh-318, HoTh-516, BP-TJ-651/20 and S-2003-US-633; while, minimum pol percentage was recorded in Co-639 (13.58±0.14%) followed by AP-04-46/02, AP-98-156/08, CP-52-28, Co-413, CP-69-1059, CoJ-84 and CP-29-120. Artificial inoculation of smut pathogen severely reduced pol% of 54 cultivars (6.01–25.06%) in which reduction was highly significant (t > 4.032). The most suffered cultivars included CP-29-120 (25.06%), followed by HoTh-550, CSSG-1741, Tritan and CoJ-84. The pol of 28 cultivars remained un-affected as a non-significant (t<2.571) reduction was found in inoculated setts compared to the un-inoculated ones. Smut pathogen moderately affected 22 cultivars in which reduction in pol percentage was ranging from 3.23 to 5.75% and (t>2.571) (S2 Table).

**Table 4:** Analysis of variance of cultivars and disease treatments for pol contents.

Source of variation	DF	SS	MS	F-Ratio
A (Treatments)	1	508.59	508.586	528.57*
Blocks (Replicates)	5	1.12	0.224	
Error A (Blocks*Treatments)	5	4.81	0.962	
B (Cultivars)	103	2766.15	26.856	141.15*
A*B (Treatments*Cultivars)	103	331.93	3.223	16.94*
Error B (Blocks*Treatments*Cultivars)	1030	195.97	0.190	
Total	1247	3808.56		

* p<0.0001.

**Fig 2:**
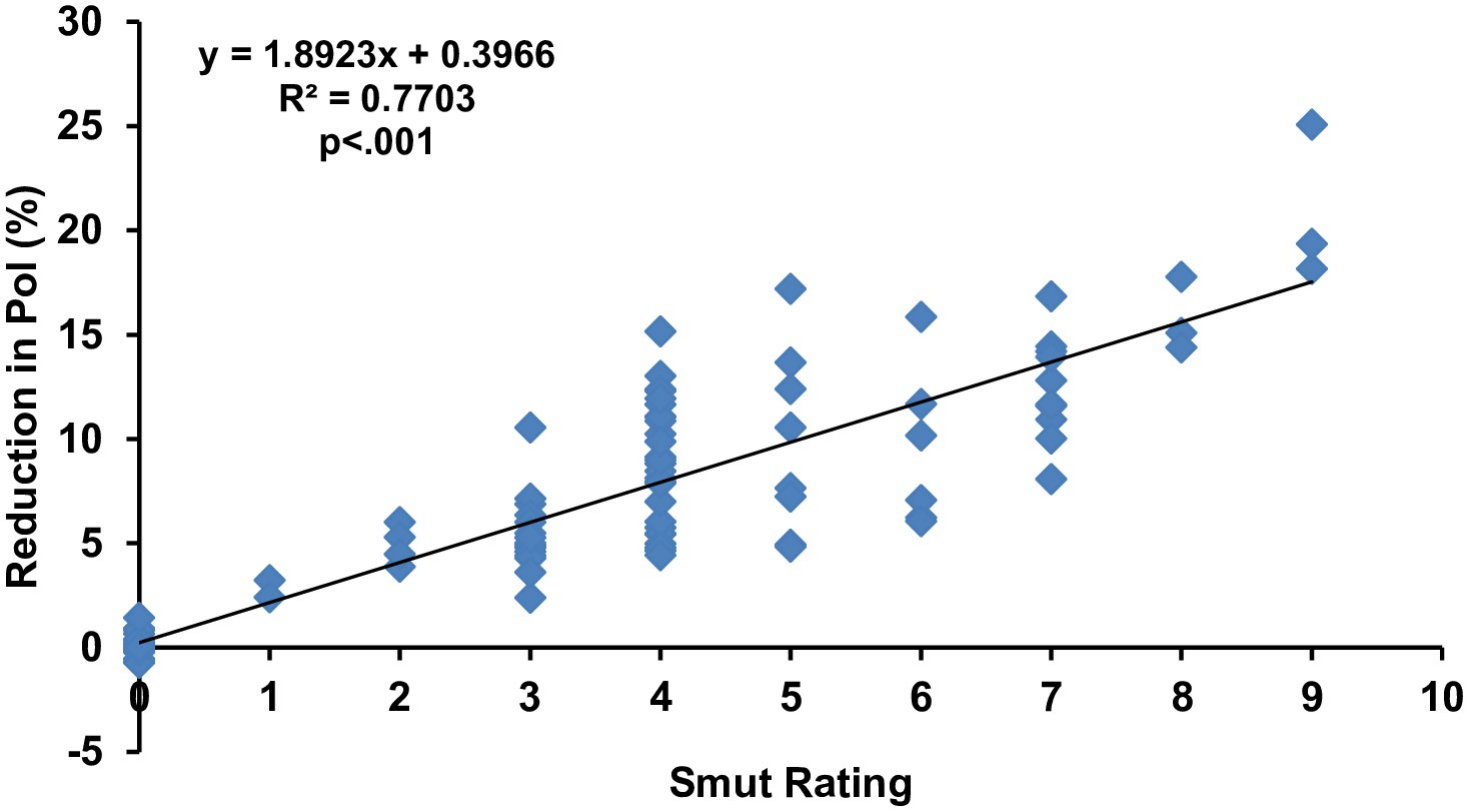
Regression analysis of pol percentage with whip smut disease rating. Note: The smut rating is not a linear scale. It’s based on the transformation explained in methods from linear incidence value to the rating. Accordingly, the resulting disease incidences were converted into 0–9 disease rating scale [41], where 0: no disease, 1: 0.1–2.5%, 2: 2.6–5.5%, 3: 5.6–7.5%, 4: 7.6–12.5%, 5: 12.6–15.5%, 6: 15.6–18.0%, 7: 18.1–22.5%, 8: 22.6–25.5% and 9: 25.6–100%.

In the case of the effect of smut rating and the reduction in the percentage of pol (Fig 2), the graph revealed that there was a positive and highly significant relationship (R^2^ = 0.7703, p<.001) between the disease and pol. The regression equation further showed that with an increase of one rating in disease may vary pol up to 1.8923%.

### Effects of whip smut *S*. *scitamineum* on fiber contents

On an overall basis, cultivars showed significant impact (DF = 103, F = 84.51, P = 0.000). Moreover, pathogen infection also adversely affected the fiber contents in susceptible cultivars. The pathogen treatment’s effect was also highly significant (DF = 1, F = 446.87, P = 0.000). The interactive effect of cultivars and treatments also appeared highly significant (DF = 103, F = 11.37, P = 0.000) (Tables 3 and 5). In plots, inoculated with the whip smut pathogen, maximum mean fiber percentage was recorded in CP-52-28 (14.95±0.16%), Co-639 (14.92±0.21%), Co-413 (14.88±0.15%), CoJ-84 (14.84±0.16%), AP-98-156/08 (14.82±0.09%) and CP-69-1059 (14.79±0.14%). The cultivars like BPTh-807, HoTh-318, HoTh-516 and BP-TJ-651/20 produced significantly less fiber content. The artificial inoculation of smut pathogen increased fiber content in 41 cultivars and caused a highly significant (t>4.032) increase of 5.09 to 13.23% more fiber as compared to uninoculated. In comparison to un-inoculated plants, a maximum increase in fiber contents was found in inoculated plants of cv. HoTh-550 (13.23%), HoTh-408 (12.72%), CP-29-120 (12.08%) and Tritan (11.81%). About 21 cultivars showed moderate reaction to smut pathogen and produced 2.86 to 5.06% increased fiber content as compared to uninoculated. However, there was no significant (t<2.571) difference in fiber recorded in 42 cultivars among inoculated and un-inoculated planting materials (S3 Table).

**Table 5:** Analysis of variance of cultivars and disease treatments for fiber contents.

Source of variation	DF	SS	MS	F-Ratio
A (Treatments)	1	111.948	111.948	446.87*
Blocks (Replicates)	5	0.568	0.114	
Error A (Blocks*Treatments)	5	1.253	0.251	
B (Cultivars)	103	656.522	6.374	84.51*
A*B (Treatments*Cultivars)	103	88.365	0.858	11.37*
Error B (Blocks*Treatments*Cultivars)	1030	77.690	0.075	
Total	1247	936.346		

* p<0.0001.

**Fig 3:**
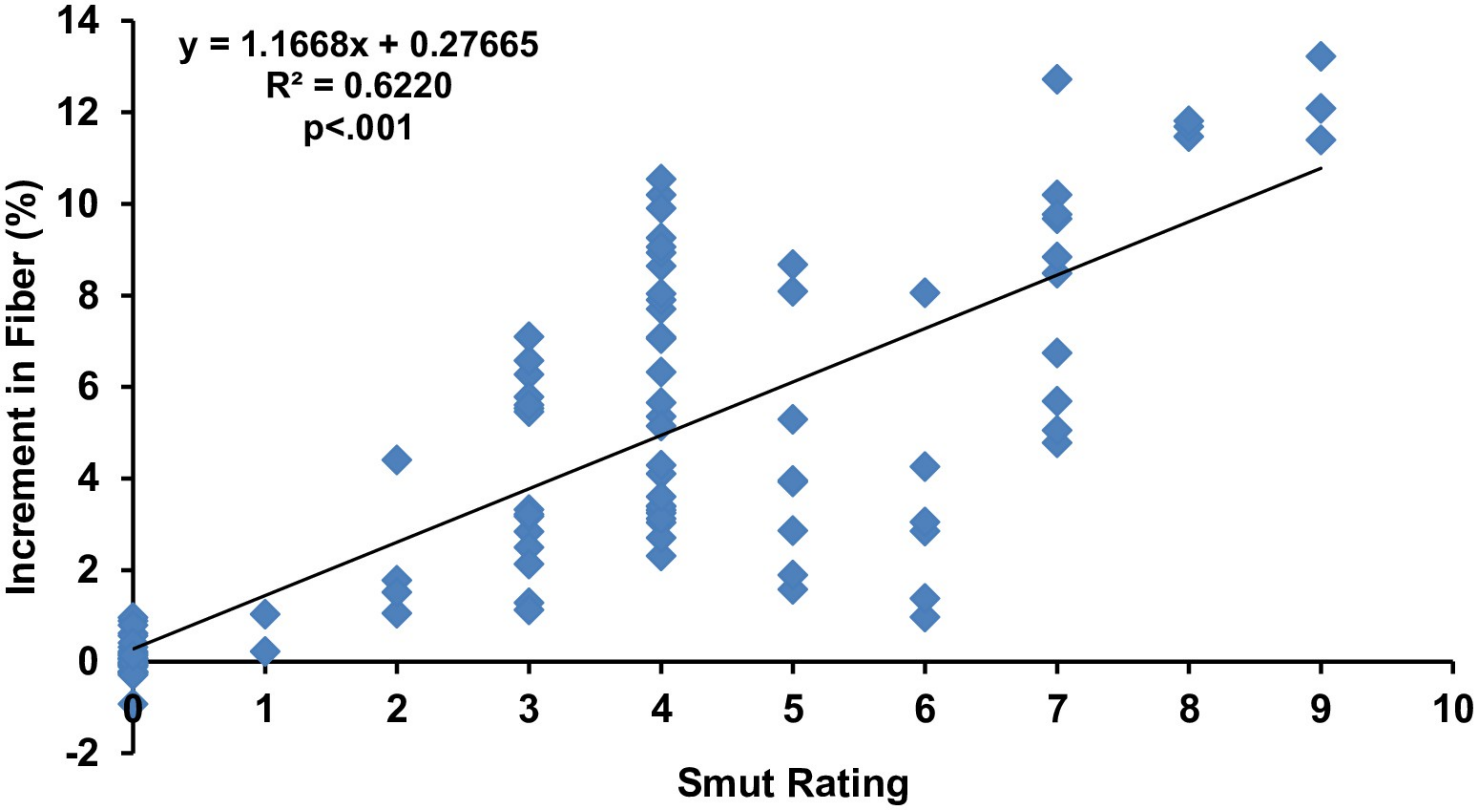
Regression analysis of fiber percentage with whip smut disease rating. Note: The smut rating is not a linear scale. It’s based on the transformation explained in methods from linear incidence value to the rating. Accordingly, the resulting disease incidences were converted into 0–9 disease rating scale [41], where 0: no disease, 1: 0.1–2.5%, 2: 2.6–5.5%, 3: 5.6–7.5%, 4: 7.6–12.5%, 5: 12.6–15.5%, 6: 15.6–18.0%, 7: 18.1–22.5%, 8: 22.6–25.5% and 9: 25.6–100%.

The regression analysis equation (y = 1.1668 x + 0.27665) in (Fig 3) for predicting the influence of smut disease rating on fiber content, showed a positive and significant (p<.001) relationship between the disease rating and fiber. The trend line and the value of R^2^ (0.6220) revealed the goodness of the regression model.

### Effects of whip smut *S*. *scitamineum* on purity

The inoculation of *S*. *scitamineum* caused a significant reduction in purity, i.e., 82.78% as compared to those of un-inoculated ones i.e., 84.40%. The overall impact of disease treatments was highly significant (DF = 1, F = 231.19, P = 0.000). The overall response of cultivars to purity was also highly significant (p<0.0001). The ‘Cultivars x Treatments’ to purity percentage was also found highly significant (DF = 103, F = 9.96, P = 0.000) (Tables 3 and 6). In plots inoculated with *S*. *scitamineum*, maximum mean purity was found in BP-TJ-651/20 (86.33±0.29%) followed by HoTh-318, HoTh-516, S-2003-US-633 and BPTh-807; while minimum purity was recorded in CP-29-120 (76.11±0.57%) followed by AP-04-46/02, AP-98- 156/08, Co-639, CP-69-1059 and Tritan. The whip smut pathogen adversely influenced the purity percentage of 41 cultivars and caused a highly significant reduction (t>4.032) ranging from 2.05 to 10.42%. The most suffered cultivars were CP-29-120 (10.42%), followed by HoTh-550 (6.77%), CSSG-1741 (5.42%), Co-208 (5.10%) and Tritan (5.06%) reduction in purity. Moreover, there was no significant difference in purity% noticed in 31 cultivars among inoculated and un-inoculated planting materials. About 32 cultivars showed moderate reaction to smut pathogen in which reduction in purity was ranging from 0.96 to 2.01% (S4 Table).

**Table 6:** Analysis of variance of cultivars and disease treatments for purity percentage.

Source of variation	DF	SS	MS	F-Ratio
A (Treatments)	1	816.74	816.741	231.19*
Blocks (Replicates)	5	28.48	5.697	
Error A (Blocks*Treatments)	5	17.66	3.533	
B (Cultivars)	103	3047.73	29.590	44.96*
A*B (Treatments*Cultivars)	103	675.13	6.555	9.96*
Error B (Blocks*Treatments*Cultivars)	1030	677.88	0.658	
Total	1247	5263.62		

* p<0.0001.

**Fig 4:**
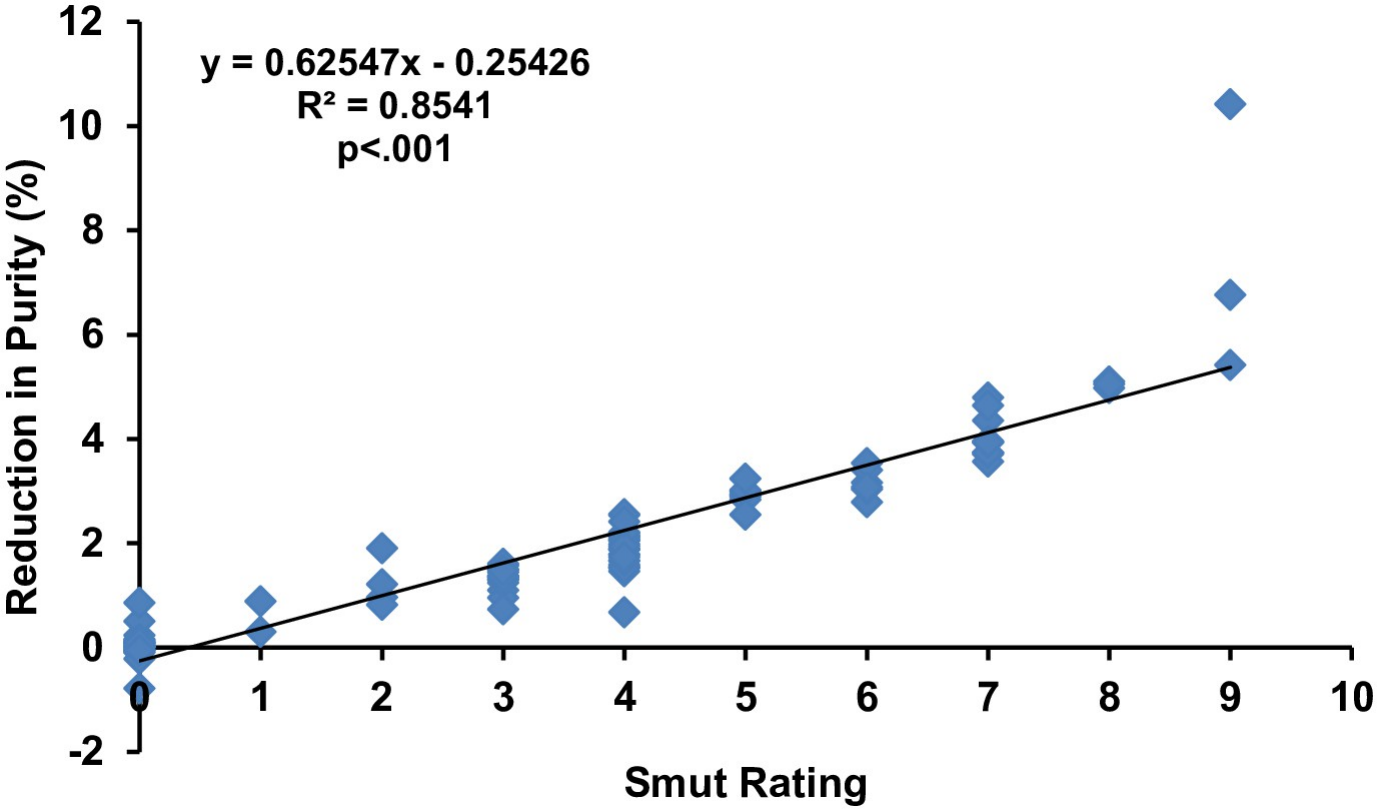
Regression analysis of purity percentage with whip smut disease rating. Note: The smut rating is not a linear scale. It’s based on the transformation explained in methods from linear incidence value to the rating. Accordingly, the resulting disease incidences were converted into 0–9 disease rating scale [41], where 0: no disease, 1: 0.1–2.5%, 2: 2.6–5.5%, 3: 5.6–7.5%, 4: 7.6–12.5%, 5: 12.6–15.5%, 6: 15.6–18.0%, 7: 18.1–22.5%, 8: 22.6–25.5% and 9: 25.6–100%.

The regression analysis results and graphical representation (Fig 4) for the impact of smut disease incidence rating on the reduction percentage of purity demonstrated that there were 85% (R^2^ = 0.8541) chances that the smut rate was responsible for the reduction in purity percentage. Not only positive (y = 0.62547x - 0.25426) but there was a highly significant relationship (p<.001) between the disease incidence and reduction percentage of purity.

### Effects of whip smut *S*. *scitamineum* on CCS

In tested sugarcane cultivars CCS percentage was greatly varied. The ANOVA showed a highly significant (p<0.0001) impact of cultivars on CSS percentage (DF = 103, F = 224.11, P = 0.000). The pathogen infection also remarkably reduced the CCS contents in most susceptible cultivars, resulting in a highly significant difference in treatments (DF = 1, F = 693.59, P = 0.000). Moreover, the interaction ‘Treatments x Cultivars’ was also highly significant (DF = 103, F = 30.11, P = 0.000) (Tables 3 and 7). In inoculated plots, maximum CCS was obtained in CP-70-530 (14.12±0.07%) and S-2003-US-633 (14.11±0.04%).; while minimum CCS had been recorded in Co-639 (9.22±0.13%) followed by AP-98-156/08, CP-29-120, AP-04-46/02, CP-69-1059 and CP-52-28. The inoculation of smut pathogen brought a highly significant (t>4.032) reduction (4.40–32.10%) in the CCS% of 66 cultivars. The maximum reduction in CCS was recorded in CP-29-120 (32.10%), followed by CSSG-1741 (21.53%), HoTh-550 (19.71%), HoTh-409 (16.92%), Tritan (16.85%) and CP-85-SP-571 (15.53%). In 27 cultivars, pathogen failed to cause significant reduction in CCS, which indicated that either they are immune, strongly resistant, or tolerant; while the remaining 11 cultivars were moderately affected with smut pathogen and showed a 1.17–4.06% reduction in CCS as compared to the un-inoculated plot of same cultivars (S5 Table).

**Table 7:** Analysis of variance of cultivars and disease treatments for CSS percentage.

Source of variation	DF	SS	MS	F-Ratio
A (Treatments)	1	235.92	235.918	693.59*
Blocks (Replicates)	5	1.37	0.275	
Error A (Blocks*Treatments)	5	1.70	0.340	
B (Cultivars)	103	1479.27	14.362	224.11*
A*B (Treatments*Cultivars)	103	198.73	1.929	30.11*
Error B (Blocks*Treatments*Cultivars)	1030	66.01	0.064	
Total	1247	1983.00		

* p<0.0001.

**Fig 5:**
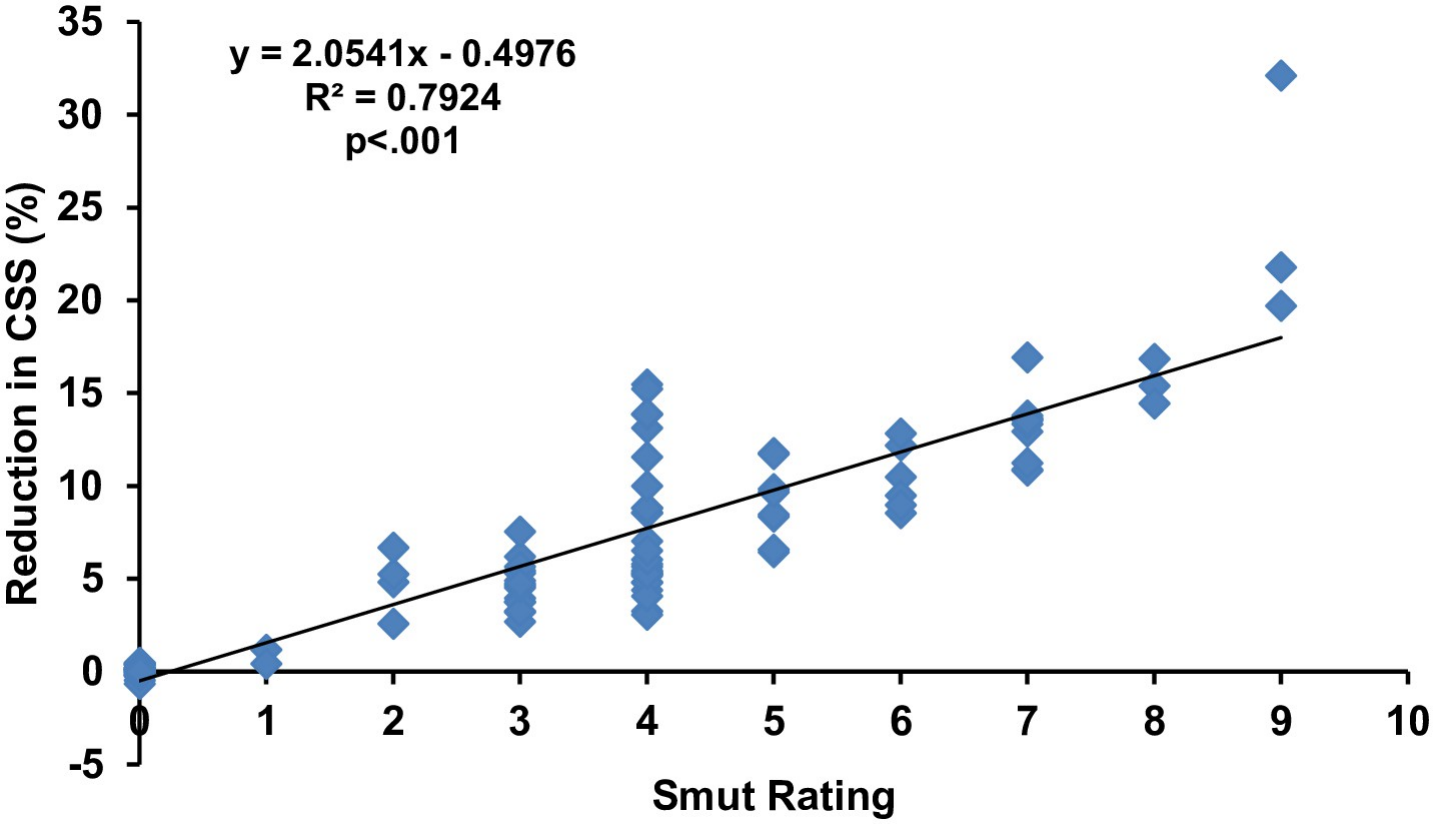
Regression analysis of brix percentage with whip smut disease rating. Note: The smut rating is not a linear scale. It’s based on the transformation explained in methods from linear incidence value to the rating. Accordingly, the resulting disease incidences were converted into 0–9 disease rating scale [41], where 0: no disease, 1: 0.1–2.5%, 2: 2.6–5.5%, 3: 5.6–7.5%, 4: 7.6–12.5%, 5: 12.6–15.5%, 6: 15.6–18.0%, 7: 18.1–22.5%, 8: 22.6–25.5% and 9: 25.6–100%.

The results regarding the relationship of the reduction percentage of CCS and intensity of disease through regression analysis (Fig 5) exhibited that the CCS of sugarcane cultivars was greatly influenced by the rating of smut disease. There was a strong (R^2^ = 0.7924) and a highly significant (p<.001) relationship between the two variables. The calculated regression equation (y = 2.0541x - 0.4976) forecast that each unit increase in smut rating causes a 2.0541 percent reduction in the CCS of sugarcane.

## Discussions

Sugarcane smut is widespread in almost all cane-growing areas of the world and considered one of the most important factors in varietal development programs. A high level of susceptibility to smut has forced to stop the commercial cultivation of many high-yielding varieties [45–47]. The infection of *S*. *scitamineum* not only reduced physical cane yield but also brought a significant reduction in the quality of cane juice, the ultimate product [24, 48]. However, the extent of losses depends upon disease severity, which is influenced by climatic factors and cultivars [21, 22, 49]. Searching of cultivars, which performed better in terms of all desirable parameters in the presence of smut pathogen, is an ongoing process. Besides quantitative traits, the impact of different diseases on juice quality parameters has been the main concern of scientists [50–53]. In the present investigation in which effects of *S*. *scitamineum* and different cultivars on qualitative parameters were assessed, it appears that pathogen treatment’s main effect, as well as cultivars main effects caused a highly significant impact on all tested quality characteristics. In aggregate, brix, pol, purity, and CSS contents significantly decreased in smut inoculated plots, while fiber tends to enhance. The regression analysis also indicates a strong and positive relationship between increased disease rating and quality parameters. In inoculated plots, cultivars produced an immune or resistant response to smut pathogen, showed no or less adverse impact on quality traits. On the other hand, in highly susceptible cultivars maximum reduction of 16.34% in brix, 25% in pol, 10.42% in purity, and 32% in CSS. While fiber contents in susceptible cultivars increased up to 13%. Many other workers also found a significant reduction in cane juice quality along with quantitative losses due to smut disease [31, 54, 55]. However, the extent of losses mainly depends upon the varieties under cultivation. Alexander [56] and Thurston [57] calculated 15–20% yield and quality losses under moderate levels of disease. More specifically, 32% losses in quality [26] and 22.2% in sucrose content were also noted in smut infected canes [58]. Our findings are in accordance with Kumar et al. [32] and Irvine [30] who also observed a significant reduction in sucrose; purity, brix, and viscosity due to smut infection. Besides whip smut, other biotic stresses such as red rot and wilt diseases also caused adverse effects on qualitative traits. In susceptible cane cultivars, wilt disease caused a reduction of 44.48% in brix, 59% in pol, 25.7% in purity, and 66% in CSS [52]. Similarly, varietal screening and use of control measures against red rot disease have been continuously carried out due to its negative impact on qualitative parameters [59–61]. In India, red rot disease caused 20.04–38.79% reduction in brix, (17.68–31.56% in purity 29.06–53.57% in CSS [51]. The findings of the present studies confirmed that sugarcane juice quality characters were greatly affected by the level of smut infection in a particular cultivar. Moreover, comprehensive field testing of cultivars objectively revealed the demonstrated the cane cultivars performed well in all qualitative parameters in the presence of smut pathogen.

## Supporting information

S1 Table Effects of whip smut *Sporisorium scitamineum* on brix percentage of sugarcane cultivars in field screening trial with artificial inoculation. (DOCX)

S2 Table Effects of whip smut *Sporisorium scitamineum* on pol percentage of sugarcane cultivars in field screening trial with artificial inoculation. (DOCX)

S3 Table Effects of whip smut *Sporisorium scitamineum* on fiber percentage of sugarcane cultivars in field screening trial with artificial inoculation. (DOCX)

S4 Table Effects of whip smut *Sporisorium scitamineum* on purity percentage of sugarcane cultivars in field screening trial with artificial inoculation. (DOCX)

S5 Table Effects of whip smut *Sporisorium scitamineum* on CCS percentage of sugarcane varieties/cultivars in field screening trial with artificial inoculation. (DOCX)
