# Supplementary material for: Impact of Sporisorium scitamineum infection on the qualitative traits of commercial cultivars and advanced lines of sugarcane
Source: PLoS One. 2022 May 23;17(5):e0268781. doi: 10.1371/journal.pone.0268781 (PMC9126389; doi:10.1371/journal.pone.0268781)
Supplement: S1 Table — (DOCX) [file pone.0268781.s001.docx]

**Table S1. Effects of whip smut *Sporisorium scitamineum* on brix percentage of sugarcane cultivars in field screening trial with artificial inoculation.**

| **S. No** | **Varieties** | **Smut**  **Rating** | **Brix (%)** | | **Reduction**  **Percent** | **T value** |
| --- | --- | --- | --- | --- | --- | --- |
|  |  |  | **Inoculated** | **Natural Infection** |  |  |
| 1 | AP-04-68/01 | 0 | 18.93±0.04^L-O^ | 18.91±0.03^MN^ | -0.09 | 0.34 |
| 2 | AP-97-56/02 | 0 | 18.28±0.07^O-R^ | 18.25±0.03^P^ | -0.19 | 0.63 |
| 3 | AP-97-69/01 | 0 | 19.90±0.08^F-J^ | 19.92±0.07^JK^ | 0.13 | -0.36 |
| 4 | AP-98-103/01 | 0 | 20.30±0.06^A-H^ | 20.36±0.04^F-H^ | 0.29 | -1.55 |
| 5 | AP-98-156/02 | 0 | 18.88±0.19^L-O^ | 19.00±0.22^MN^ | 0.65 | -0.44 |
| 6 | AP-98-156/03 | 0 | 20.30±0.10^A-H^ | 20.29±0.05^GH^ | -0.03 | 0.05 |
| 7 | AP-98-156/04 | 0 | 21.25±0.08^o-x^ | 21.24±0.03^AB^ | -0.05 | 0.19 |
| 8 | AP-98-156/07 | 0 | 19.86±0.09^G-J^ | 19.91±0.03^JK^ | 0.25 | -0.82 |
| 9 | AP-97-56/03 | 0 | 20.23±0.04^C-H^ | 20.20±0.02^H-J^ | -0.13 | 0.83 |
| 10 | BPTh-807 | 0 | 23.31±0.08^a^ | 23.23±0.05^ab^ | -0.35 | 1.57 |
| 11 | BP-TJ-651/18 | 0 | 19.39±0.08^J-L^ | 19.39±0.06^i^ | 0.04 | -0.13 |
| 12 | BP-TJ-651/20 | 0 | 22.96±0.23^a-d^ | 23.18±0.25^a-d^ | 0.97 | -0.62 |
| 13 | CB-2919 | 0 | 18.86±0.13^l-o^ | 18.75±0.08^NO^ | -0.59 | 1.00 |
| 14 | CP-70-530 | 0 | 22.74±0.07^a-e^ | 22.81±0.06^g-l^ | 0.32 | -0.70 |
| 15 | HoTh-318 | 0 | 23.23±0.07^ab^ | 23.25±0.02^ab^ | 0.11 | -0.41 |
| 16 | HoTh-4140 | 0 | 22.40±0.25^d-i^ | 22.53±0.28^kq^ | 0.60 | -0.45 |
| 17 | HoTh-438 | 0 | 22.55±0.09^b-g^ | 22.49±0.08^l-r^ | -0.27 | 1.21 |
| 18 | HoTh-516 | 0 | 23.20±0.08^a-c^ | 23.18±0.07^a-d^ | -0.09 | 0.24 |
| 19 | HoTh-544 | 0 | 20.79±0.05^v-z,A-E^ | 20.76±0.07^DE^ | -0.15 | 0.30 |
| 20 | HoTh-610 | 0 | 21.61±0.06^k-t^ | 21.58±0.04^yz^ | -0.15 | 0.67 |
| 21 | QSG-1741 | 0 | 21.72±0.02^i-q^ | 21.68±0.08^x-z^ | -0.19 | 0.47 |
| 22 | Roc-16 | 0 | 22.12±0.12^e-m^ | 21.96±0.23^u-x^ | -0.73 | 0.72 |
| 23 | S-2003-QSSG-776 | 0 | 22.51±0.19^c-h^ | 22.65±0.21^i-o^ | 0.63 | -0.60 |
| 24 | S-2003-US-633 | 0 | 22.74±0.17^a-e^ | 22.77±0.24^h-m^ | 0.13 | -0.08 |
| 25 | S-2006-SP-30 | 0 | 22.61±0.09^a-f^ | 22.56±0.08^j-q^ | -0.22 | 1.20 |
| 26 | Th-704 | 0 | 21.87±0.04^g-o^ | 21.89±0.04^v-y^ | 0.06 | -0.44 |
| 27 | AP-04-46/03 | 1 | 20.44±0.13^z,A-G^ | 20.94±0.27^B-D^ | 2.36 | -2.83^*^ |
| 28 | HoTh-344 | 1 | 21.57±0.06^l-u^ | 21.65±0.01^x-z^ | 0.37 | -1.79 |
| 29 | AP-98-156/06 | 2 | 19.33±0.23^J-M^ | 20.37±0.12^F-H^ | 5.12 | -4.11^**^ |
| 30 | CP-82-2083 | 2 | 20.19±0.15^D-H^ | 20.82±0.14^C-E^ | 3.06 | -2.79^*^ |
| 31 | HoTh-518 | 2 | 22.37±0.22^d-i^ | 23.17±0.20^a-d^ | 3.45 | -2.87^*^ |
| 32 | S-2002-HSG-200 | 2 | 20.42±0.22^A-G^ | 21.12±0.28 ^bc^ | 3.31 | -2.78^*^ |
| 33 | AP-04-59/02 | 3 | 21.33±0.18^n-w^ | 22.07±0.23^t-w^ | 3.35 | -2.80^*^ |
| 34 | AP-04-68/03 | 3 | 22.81±0.19^a-e^ | 23.13±0.01^a-g^ | 1.36 | -1.60 |
| 35 | AP-04-59/03 | 3 | 17.76±0.13^Q-T^ | 18.42±0.15^OP^ | 3.59 | -3.07^*^ |
| 36 | BPTh-804 | 3 | 21.75±0.46^i-q^ | 23.04±0.41^c-j^ | 5.60 | -4.76^**^ |
| 37 | CPS-1827 | 3 | 21.65±0.20^j-r^ | 22.94±0.22^a-i^ | 5.65 | -4.92^**^ |
| 38 | Chandka | 3 | 21.50±0.16^m-u^ | 22.82±0.12^f-l^ | 5.75 | -5.12^**^ |
| 39 | CO-620 | 3 | 21.16±0.31^p-y^ | 22.18±0.28^r-v^ | 4.58 | -3.39^*^ |
| 40 | CPSG-244-S-2083 | 3 | 22.40±0.16^d-i^ | 23.24±0.19^ab^ | 3.61 | -3.48^*^ |
| 41 | HoTh-419 | 3 | 21.98±0.19^f-n^ | 22.64±0.16^i-o^ | 2.89 | -2.64^*^ |
| 42 | HoTh-424 | 3 | 22.33±0.19^d-j^ | 23.14±0.08^a-g^ | 3.51 | -3.17^*^ |
| 43 | HoTh-513 | 3 | 21.58±0.25^l-u^ | 22.51±0.10^k-r^ | 4.17 | -3.55^*^ |
| 44 | HoTh-517 | 3 | 22.53±0.21^b-h^ | 23.16±0.14^a-d^ | 2.73 | -2.94^*^ |
| 45 | S-2003-HOSG-679 | 3 | 22.59±0.23^b-f^ | 23.13±0.09^a-g^ | 2.36 | -2.69^*^ |
| 46 | S-2003-US-160 | 3 | 21.47±0.30^m-v^ | 22.35±0.06^o-t^ | 3.96 | -2.98^*^ |
| 47 | Th-720 | 3 | 18.76±0.31^L-O^ | 20.66±0.08^D-F^ | 9.21 | -6.42^**^ |
| 48 | AP-04-68/02 | 4 | 21.83±0.22^h-p^ | 22.61±0.10^i-p^ | 3.44 | -2.62^*^ |
| 49 | B-43405 | 4 | 21.57±0.32^l-u^ | 22.48±0.10^m-r^ | 4.03 | -3.26^*^ |
| 50 | B-46364 | 4 | 20.94±0.26^s-z,AB^ | 22.44±0.11^m-s^ | 6.70 | -4.47^**^ |
| 51 | BP-TJ-15/01 | 4 | 21.62±0.35^k-s^ | 23.20±0.28^a-d^ | 6.80 | -4.56^**^ |
| 52 | CPF-229 | 4 | 21.45±0.32^m-v^ | 22.93±0.11^b-i^ | 6.47 | -4.25^**^ |
| 53 | CO-413 | 4 | 17.35±0.23^ST^ | 19.93±0.28^I-K^ | 12.93 | -7.44^**^ |
| 54 | CP-52-28 | 4 | 17.12±0.19^T^ | 19.13±0.11^LM^ | 10.53 | -6.83^**^ |
| 55 | CP-70-SP-1215 | 4 | 18.50±0.38^N-P^ | 20.28±0.07^GH^ | 8.78 | -5.76^**^ |
| 56 | CP-85-SP-571 | 4 | 19.27±0.32^J-M^ | 21.73±0.06^x-z^ | 11.36 | -7.40^**^ |
| 57 | CSSG-2402 | 4 | 20.47±0.35^yz,A-G^ | 22.74±0.06^h-n^ | 9.99 | -6.15^**^ |
| 58 | CSSG-2476 | 4 | 22.30±0.23^d-k^ | 23.14±0.11^a-g^ | 3.65 | -2.99^*^ |
| 59 | H-86-NSG-311 | 4 | 20.42±0.45^A-G^ | 22.46±0.18^m-r^ | 9.08 | -6.33^**^ |
| 60 | HoTh-316 | 4 | 22.36±0.27^d-i^ | 23.19±0.04^a-d^ | 3.56 | -2.90^*^ |
| 61 | HoTh-127 | 4 | 17.83±0.27^P-S^ | 19.85±0.06^K^ | 10.15 | -6.78^**^ |
| 62 | HoTh-326 | 4 | 21.63±0.30^j-s^ | 22.67±0.06^i-o^ | 4.57 | -3.45^*^ |
| 63 | HoTh-432 | 4 | 22.48±0.23^d-h^ | 23.07±0.11^a-h^ | 2.54 | -2.63^*^ |
| 64 | HoTh-518 | 4 | 21.65±0.38^j-r^ | 22.57±0.13^j-q^ | 4.08 | -3.32^*^ |
| 65 | HoTh-612 | 4 | 22.52±0.21^b-h^ | 23.21±0.06^a-c^ | 2.95 | -2.67^*^ |
| 66 | NSG-60 | 4 | 18.42±0.29^N-Q^ | 20.26±0.03^G-I^ | 9.06 | -5.90^**^ |
| 67 | Q-88 | 4 | 21.65±0.29^j-r^ | 22.87±0.03^d-j^ | 5.31 | -4.22^**^ |
| 68 | S-2003-CPSG-704 | 4 | 20.95±0.22^r-z,AB^ | 22.28±0.05^p-u^ | 5.98 | -5.27^**^ |
| 69 | S-2006-SP-18 | 4 | 21.29±0.22^n-w^ | 22.92±0.10^b-i^ | 7.10 | -5.38^**^ |
| 70 | S-2003-CPSG-193 | 4 | 21.72±0.30^i-q^ | 23.15±0.03^a-f^ | 6.16 | -5.10^**^ |
| 71 | SPSG-3481 | 4 | 20.59±0.22^x-z,A-F^ | 22.70±0.25^i-n^ | 9.30 | -6.37^**^ |
| 72 | Th-702 | 4 | 21.13±0.28^q-z^ | 22.83±0.04^e-k^ | 7.45 | -5.59^**^ |
| 73 | Th-725 | 4 | 18.48±0.29^n-p^ | 20.63±0.06^D-F^ | 10.41 | -6.77^**^ |
| 74 | Th-10 | 4 | 21.39±0.29^n-v^ | 23.16±0.02^a-e^ | 7.64 | -5.80^**^ |
| 75 | AP-04-46/02 | 5 | 17.85±0.24^P-S^ | 18.78±0.04^N^ | 4.91 | -3.91^*^ |
| 76 | COJ-84 | 5 | 17.54±0.34^ST^ | 20.55±0.09^E-G^ | 14.65 | -10.00^**^ |
| 77 | CP-75-1353 | 5 | 19.09±0.31^K-N^ | 21.16±0.03^B^ | 9.77 | -6.66^**^ |
| 78 | HoTh-401 | 5 | 22.60±0.20^b-f^ | 23.07±0.03^a-h^ | 2.05 | -2.59^*^ |
| 79 | HSF-240 | 5 | 19.33±0.38^J-M^ | 21.78±0.12^w-z^ | 11.17 | -6.95^**^ |
| 80 | NCO-310 | 5 | 19.33±0.28^J-M^ | 20.96±0.03^B-D^ | 7.77 | -5.68^**^ |
| 81 | S-2003-US-704 | 5 | 22.22±0.27^e-l^ | 23.17±0.06^a-d^ | 4.15 | -3.59^*^ |
| 82 | S-2006-SP-658 | 5 | 21.43±0.21^m-v^ | 21.96±0.04^u-x^ | 2.42 | -2.85^*^ |
| 83 | AP-98-156/05 | 6 | 18.45±0.19^N-Q^ | 19.06±0.03^MN^ | 3.16 | -2.90^*^ |
| 84 | AP-04-59/01 | 6 | 21.53±0.33^l-u^ | 23.15±0.05^a-e^ | 7.01 | -5.27^**^ |
| 85 | AP-98-156/08 | 6 | 17.60±0.24^R-T^ | 18.41±0.03^P^ | 4.41 | -3.11^*^ |
| 86 | CO-639 | 6 | 17.24±0.33^ST^ | 19.76±0.04^K^ | 12.77 | -7.24^**^ |
| 87 | S-2003-HOSG-1626 | 6 | 22.40±0.20^d-i^ | 23.13±0.08^a-g^ | 3.13 | -2.86^*^ |
| 88 | YT-236 | 6 | 18.54±0.34^N-P^ | 20.34±0.06^F-H^ | 8.87 | -5.85^**^ |
| 89 | AP-98-156/01 | 7 | 20.65±0.36^w-z,A-E^ | 22.48±0.03^l-r^ | 8.17 | -5.45^**^ |
| 90 | CO-1148 | 7 | 21.60±0.34^k-t^ | 22.62±0.06^i-o^ | 4.50 | -3.33^*^ |
| 91 | COJ-81 | 7 | 18.94±0.25^L-O^ | 20.51±0.08^E-H^ | 7.66 | -5.46^**^ |
| 92 | CP-59-1059 | 7 | 21.22±0.33^o-x^ | 23.27±0.04^a^ | 8.83 | -5.78^**^ |
| 93 | CP-69-1059 | 7 | 17.71±0.32^R-T^ | 19.92±0.02^JK^ | 11.10 | -7.23^**^ |
| 94 | HoTh-408 | 7 | 20.25±0.41^B-H^ | 23.18±0.06^a-d^ | 12.65 | -7.49^**^ |
| 95 | HoTh-409 | 7 | 19.24±0.29^J-M^ | 21.54±0.07^z,A^ | 10.66 | -6.95^**^ |
| 96 | Larkana-2001 | 7 | 21.00±0.32^r-z,A^ | 22.42±0.06^n-s^ | 6.33 | -4.18^**^ |
| 97 | S-2002-SFSD-1307 | 7 | 20.34±0.33^A-H^ | 22.11±0.04^s-v^ | 8.02 | -5.34^**^ |
| 98 | S-2003-HOSG-701 | 7 | 20.91±0.36^t-z,A-C^ | 23.17±0.05^a-d^ | 9.75 | -6.14^**^ |
| 99 | CO-208 | 8 | 20.76±0.41^v-z,A-Z^ | 23.21±0.06^a-c^ | 10.54 | -6.67^**^ |
| 100 | CPD-01-359 | 8 | 20.89±0.39 ^u-z,A-D^ | 23.18±0.04^a-d^ | 9.91 | -6.20^**^ |
| 101 | Tritan | 8 | 19.70±0.41^H-K^ | 22.74±0.06^h-n^ | 13.39 | -8.13^**^ |
| 102 | CP-29-120 | 9 | 18.64±0.33^M-O^ | 22.28±0.05^q-u^ | 16.34 | -11.06^**^ |
| 103 | CSSG-1741 | 9 | 19.42±0.37^I-L^ | 22.44±0.12^m-r^ | 13.47 | -8.28^**^ |
| 104 | HoTh-550 | 9 | 20.11±0.39^E-I^ | 23.25±0s.05^ab^ | 13.52 | -8.54^**^ |
|  | F-Statistics at df = 103 | | 40.78 | 144.83 |  |  |
|  | LSD 0.05 | | 0.7053 | 0.3319 |  |  |

ns= Non-significant at 0.05, * = significant at 0.05; and ** = highly significant at 0.01 level

Means followed by same letter(s) in the same column are not significantly different at 0.05 LSD.
