# Supplementary material for: Impact of Sporisorium scitamineum infection on the qualitative traits of commercial cultivars and advanced lines of sugarcane
Source: PLoS One. 2022 May 23;17(5):e0268781. doi: 10.1371/journal.pone.0268781 (PMC9126389; doi:10.1371/journal.pone.0268781)
Supplement: S2 Table — (DOCX) [file pone.0268781.s002.docx]

**Table S2. Effects of whip smut *Sporisorium scitamineum* on pol percentage of sugarcane cultivars in field screening trial with artificial inoculation.**

| **S. No** | **Varieties** | **Smut**  **Rating** | **pol (%)** | | **Reduction**  **Percent** | **T value** |
| --- | --- | --- | --- | --- | --- | --- |
|  |  |  | **Inoculated** | **Natural Infection** |  |  |
| 1 | AP-04-68/01 | 0 | 15.50±0.16^E-K^ | 15.48±0.20^q^ | -0.08 | 0.04 |
| 2 | AP-97-56/02 | 0 | 14.89±0.02^KL^ | 14.87±0.01r | -0.09 | 0.67 |
| 3 | AP-97-69/01 | 0 | 16.50±0.16^yz,A-C^ | 16.39±0.16^mn^ | -0.65 | 0.67 |
| 4 | AP-98-103/01 | 0 | 16.91±0.03^v-z^ | 16.95±0.01^l^ | 0.24 | -1.81 |
| 5 | AP-98-156/02 | 0 | 15.70±0.19^D-H^ | 15.80±0.16^p^ | 0.65 | -0.59 |
| 6 | AP-98-156/03 | 0 | 16.94±0.02^v-z^ | 16.93±0.01^l^ | -0.07 | 0.34 |
| 7 | AP-98-156/04 | 0 | 18.03±0.01^k-p^ | 18.04±0.01^i^ | 0.03 | -0.37 |
| 8 | AP-98-156/07 | 0 | 16.47±0.12^z,A-C^ | 16.49±0.15^m^ | 0.16 | -0.37 |
| 9 | AP-97-56/03 | 0 | 16.91±0.02^v-z^ | 16.91±0.01^l^ | 0.01 | -0.09 |
| 10 | BPTh-807 | 0 | 20.04±0.03^a^ | 20.02±0.01^a^ | -0.11 | 0.85 |
| 11 | BP-TJ-651/18 | 0 | 16.04±0.03^B-E^ | 16.05±0.02^op^ | 0.06 | -0.42 |
| 12 | BP-TJ-651/20 | 0 | 19.82±0.08^ab^ | 20.00±0.01^a^ | 0.92 | -2.26 |
| 13 | CB-2919 | 0 | 15.38±0.09^F-K^ | 15.30±0.21^q^ | -0.54 | 0.41 |
| 14 | CP-70-530 | 0 | 19.18±0.07^b-f^ | 19.35±0.20^b-d^ | 0.84 | -0.75 |
| 15 | HoTh-318 | 0 | 20.02±0.02^a^ | 20.03±0.00^a^ | 0.02 | -0.20 |
| 16 | HoTh-4140 | 0 | 18.95±0.29^c-i^ | 19.02±0.50^ef^ | 0.34 | -0.13 |
| 17 | HoTh-438 | 0 | 19.02±0.03^c-i^ | 18.98±0.02^ef^ | -0.22 | 2.06 |
| 18 | HoTh-516 | 0 | 20.00±0.02^a^ | 20.01±0.02^a^ | 0.07 | -0.51 |
| 19 | HoTh-544 | 0 | 17.07±0.02^u-z^ | 17.05±0.02^l^ | -0.12 | 0.74 |
| 20 | HoTh-610 | 0 | 18.13±0.05^k-n^ | 18.12±0.01^i^ | -0.07 | 0.31 |
| 21 | QSG-1741 | 0 | 18.16±0.02^j-n^ | 18.15±0.02^hi^ | -0.09 | 0.45 |
| 22 | Roc-16 | 0 | 18.67±0.10^e-k^ | 18.53±0.15^g^ | -0.72 | 0.94 |
| 23 | S-2003-QSSG-776 | 0 | 18.94±0.03^d-i^ | 19.01±0.01^ef^ | 0.41 | -2.28 |
| 24 | S-2003-US-633 | 0 | 19.58±0.22^a-c^ | 19.86±0.17^a^ | 1.44 | -1.41 |
| 25 | S-2006-SP-30 | 0 | 19.02±0.04^c-i^ | 18.99±0.02^ef^ | -0.13 | 1.01 |
| 26 | Th-704 | 0 | 18.40±0.14^i-m^ | 18.41±0.14^gh^ | 0.01 | -0.02 |
| 27 | AP-04-46/03 | 1 | 17.10±0.30^t-z^ | 17.67±0.20^j^ | 3.23 | -2.66^*^ |
| 28 | HoTh-344 | 1 | 18.10±0.32^k-o^ | 18.55±0.17^g^ | 2.41 | -2.28 |
| 29 | AP-98-156/06 | 2 | 15.93±0.23^C-F^ | 16.95±0.03^l^ | 6.03 | -4.12^**^ |
| 30 | CP-82-2083 | 2 | 16.69±0.10^x-z,A^ | 17.36±0.17^k^ | 3.87 | -2.79^*^ |
| 31 | HoTh-518 | 2 | 18.95±0.19^c-i^ | 20.01±0.11^a^ | 5.31 | -3.62^*^ |
| 32 | S-2002-HSG-200 | 2 | 17.20±0.24^s-x^ | 18.01±0.01^i^ | 4.48 | -3.26^*^ |
| 33 | AP-04-59/02 | 3 | 18.04±0.26^k-p^ | 18.88±0.01^f^ | 4.42 | -3.16^*^ |
| 34 | AP-04-68/03 | 3 | 19.52±0.24^a-d^ | 19.99±0.06^a^ | 2.31 | -2.26 |
| 35 | AP-04-59/03 | 3 | 14.23±0.23^MN^ | 14.94±0.03^r^ | 4.78 | -3.36^*^ |
| 36 | BPTh-804 | 3 | 18.47±0.24^g-m^ | 19.83±0.15^a^ | 6.87 | -4.82^**^ |
| 37 | CPS-1827 | 3 | 18.13±0.20^k-n^ | 19.52±0.20^b^ | 7.15 | -4.94^**^ |
| 38 | Chandka | 3 | 17.85±0.23^m-r^ | 19.06±0.10^ef^ | 6.36 | -4.57^**^ |
| 39 | CO-620 | 3 | 17.76±0.29^n-s^ | 18.90±0.01^f^ | 6.01 | -4.04^**^ |
| 40 | CPSG-244-S-2083 | 3 | 19.05±0.24^c-h^ | 20.02±0.12^a^ | 4.88 | -3.39^*^ |
| 41 | HoTh-419 | 3 | 18.14±0.37^k-n^ | 19.01±0.10^ef^ | 4.16 | -3.10^*^ |
| 42 | HoTh-424 | 3 | 19.00±0.24^c-i^ | 20.00±0.09^a^ | 4.87 | -3.39^*^ |
| 43 | HoTh-513 | 3 | 17.94±0.24^l-q^ | 18.98±0.08^ef^ | 5.49 | -3.97^*^ |
| 44 | HoTh-517 | 3 | 19.15±0.27^c-f^ | 20.01±0.16^a^ | 4.29 | -3.23^*^ |
| 45 | S-2003-HOSG-679 | 3 | 19.27±0.21^b-e^ | 20.00±0.04^a^ | 3.63 | -2.83^*^ |
| 46 | S-2003-US-160 | 3 | 17.95±0.25^l-q^ | 18.95±0.06^ef^ | 5.28 | -3.91^*^ |
| 47 | Th-720 | 3 | 15.22±0.23^G-K^ | 17.02±0.07^l^ | 10.55 | -7.61^**^ |
| 48 | AP-04-68/02 | 4 | 17.98±0.27^l-q^ | 19.01±0.02^ef^ | 5.44 | -3.82^*^ |
| 49 | B-43405 | 4 | 17.89±0.28^m-q^ | 18.98±0.01^ef^ | 5.75 | -3.96^*^ |
| 50 | B-46364 | 4 | 17.43±0.27^p-v^ | 18.97±0.02^ef^ | 8.13 | -5.59^**^ |
| 51 | BP-TJ-15/01 | 4 | 18.25±0.23^j-n^ | 20.02±0.15^a^ | 8.83 | -6.96^**^ |
| 52 | CPF-229 | 4 | 17.74±0.40^n-t^ | 19.38±0.14^bc^ | 8.46 | -5.98^**^ |
| 53 | CO-413 | 4 | 14.01±0.18^M-O^ | 16.51±0.09^m^ | 15.15 | -10.13^**^ |
| 54 | CP-52-28 | 4 | 14.00±0.23^M-O^ | 15.98±0.01^op^ | 12.37 | -8.36^**^ |
| 55 | CP-70-SP-1215 | 4 | 15.20±0.25^G-K^ | 16.93±0.01^l^ | 10.23 | -7.08^**^ |
| 56 | CP-85-SP-571 | 4 | 15.79±0.24^D-G^ | 18.15±0.02^hi^ | 13.02 | -9.57^**^ |
| 57 | CSSG-2402 | 4 | 16.70±0.24^x-z,A^ | 19.04±0.12^ef^ | 12.30 | -7.70^**^ |
| 58 | CSSG-2476 | 4 | 18.91±0.28^d-i^ | 20.00±0.13^a^ | 5.47 | -3.53^*^ |
| 59 | H-86-NSG-311 | 4 | 16.91±0.24^v-z^ | 18.97±0.03^ef^ | 10.87 | -7.86^**^ |
| 60 | HoTh-316 | 4 | 19.02±0.29^c-i^ | 20.01±0.01^a^ | 4.98 | -3.44^*^ |
| 61 | HoTh-127 | 4 | 14.51±0.21^LM^ | 16.42±0.18^m^ | 11.66 | -7.28^**^ |
| 62 | HoTh-326 | 4 | 17.87±0.28^m-q^ | 19.02±0.01^ef^ | 6.05 | -4.09^**^ |
| 63 | HoTh-432 | 4 | 19.06±0.19^c-g^ | 19.98±0.11^a^ | 4.65 | -3.35^*^ |
| 64 | HoTh-518 | 4 | 18.10±0.25^k-o^ | 19.00±0.03^ef^ | 4.73 | -3.45^*^ |
| 65 | HoTh-612 | 4 | 19.13±0.27^c-f^ | 20.02±0.04^a^ | 4.44 | -3.19^*^ |
| 66 | NSG-60 | 4 | 15.05±0.24^I-L^ | 16.92±0.01^l^ | 11.07 | -7.57^**^ |
| 67 | Q-88 | 4 | 17.74±0.27^n-t^ | 19.07±0.01^d-f^ | 7.00 | -4.83^**^ |
| 68 | S-2003-CPSG-704 | 4 | 17.43±0.30^p-v^ | 18.93±0.01^ef^ | 7.90 | -5.21^**^ |
| 69 | S-2006-SP-18 | 4 | 17.34±0.23^q-w^ | 19.09±0.02^d-f^ | 9.13 | -7.16^**^ |
| 70 | S-2003-CPSG-193 | 4 | 18.41±0.30^h-m^ | 20.00± 0.01^a^ | 7.95 | -5.44^**^ |
| 71 | SPSG-3481 | 4 | 16.92±0.21^v-z^ | 19.03±0.07^ef^ | 11.06 | -7.53^**^ |
| 72 | Th-702 | 4 | 17.48±0.18^o-v^ | 19.20±0.15^c-e^ | 8.99 | -6.93^**^ |
| 73 | Th-725 | 4 | 14.98±0.30^J-L^ | 17.01±0.01^l^ | 11.95 | -6.73^**^ |
| 74 | Th-10 | 4 | 18.03±0.29^l-p^ | 20.00±0.11^a^ | 9.87 | -6.67^**^ |
| 75 | AP-04-46/02 | 5 | 13.85±0.18^NO^ | 15.00±0.06^r^ | 7.66 | -5.12^**^ |
| 76 | COJ-84 | 5 | 14.07±0.24^M-O^ | 16.99±0.02^l^ | 17.20 | -11.36^**^ |
| 77 | CP-75-1353 | 5 | 15.78±0.26^D-G^ | 18.02±0.01^i^ | 12.40 | -8.48^**^ |
| 78 | HoTh-401 | 5 | 19.00±0.29^c-i^ | 19.98±0.01^a^ | 4.93 | -3.41^*^ |
| 79 | HSF-240 | 5 | 15.68±0.26^D-I^ | 18.17±0.04^hi^ | 13.68 | -8.56^**^ |
| 80 | NCO-310 | 5 | 15.81±0.37^D-G^ | 17.68±0.19^j^ | 10.56 | -780^**^ |
| 81 | S-2003-US-704 | 5 | 18.56±0.27^f-l^ | 20.01±0.02^a^ | 7.25 | -5.23^**^ |
| 82 | S-2006-SP-658 | 5 | 17.64±0.20^n-u^ | 18.54±0.15^g^ | 4.96 | -3.25^*^ |
| 83 | AP-98-156/05 | 6 | 14.97±0.23^J-L^ | 15.96±0.01^op^ | 6.22 | -4.37^**^ |
| 84 | AP-04-59/01 | 6 | 17.97±0.29^l-q^ | 20.00±0.01^a^ | 10.16 | -6.97^**^ |
| 85 | AP-98-156/08 | 6 | 13.86±0.21^NO^ | 14.91±0.01^r^ | 7.07 | -4.86^**^ |
| 86 | CO-639 | 6 | 13.58±0.14^O^ | 16.13±0.01^no^ | 15.86 | -9.18^**^ |
| 87 | S-2003-HOSG-1626 | 6 | 18.79±0.27^e-j^ | 20.00±0.02^a^ | 6.07 | -4.24^**^ |
| 88 | YT-236 | 6 | 14.96±0.26^J-L^ | 16.94±0.02^l^ | 11.68 | -7.41^**^ |
| 89 | AP-98-156/01 | 7 | 16.78±0.30^w-z,A^ | 18.98±0.01^ef^ | 11.58 | -7.20^**^ |
| 90 | CO-1148 | 7 | 17.48±0.28^o-v^ | 19.01±0.01^ef^ | 8.07 | -5.55^**^ |
| 91 | COJ-81 | 7 | 15.13±0.25^H-L^ | 16.99±0.02^l^ | 10.94 | -7.51^**^ |
| 92 | CP-59-1059 | 7 | 17.47±0.21^o-v^ | 20.03±0.11^a^ | 12.80 | -8.50^**^ |
| 93 | CP-69-1059 | 7 | 14.03±0.25^M-O^ | 16.40±0.15^mn^ | 14.43 | -8.94^**^ |
| 94 | HoTh-408 | 7 | 16.64±0.26^x-z,AB^ | 20.01±0.11^a^ | 16.84 | -10.87^**^ |
| 95 | HoTh-409 | 7 | 15.54±0.25^D-J^ | 18.11±0.06^i^ | 14.19 | -8.65^**^ |
| 96 | Larkana-2001 | 7 | 17.06±0.29^u-z^ | 18.96±0.02^ef^ | 10.01 | -6.80^**^ |
| 97 | S-2002-SFSD-1307 | 7 | 16.69±0.24^x-z,A^ | 18.89±0.06^f^ | 11.65 | -7.39^**^ |
| 98 | S-2003-HOSG-701 | 7 | 17.22±0.34^r-x^ | 20.01±0.01^a^ | 13.94 | -8.11^**^ |
| 99 | CO-208 | 8 | 17.00±0.36 ^v-z^ | 20.01±0.11^a^ | 15.08 | -9.32^**^ |
| 100 | CPD-01-359 | 8 | 17.13±0.29^s-y^ | 20.01±0.06^a^ | 14.39 | -8.86^**^ |
| 101 | Tritan | 8 | 15.65±0.33^D-I^ | 19.04±0.11^ef^ | 17.77 | -11.76^**^ |
| 102 | CP-29-120 | 9 | 14.18±0.25^M-O^ | 18.93±0.07^ef^ | 25.06 | -16.26^**^ |
| 103 | CSSG-1741 | 9 | 15.52±0.26^D-K^ | 18.97±0.03^ef^ | 18.15 | -12.50^**^ |
| 104 | HoTh-550 | 9 | 16.15±0.34^A-D^ | 20.02±0.09^a^ | 19.35 | -11.90^**^ |
|  | F-Statistics at df = 103 | | 52.64 | 211.25 |  |  |
|  | LSD 0.05 | | 0.6388 | 0.2855 |  |  |

ns= Non-significant at 0.05, * = significant at 0.05; and ** = highly significant at 0.01 level

Means followed by same letter(s) in the same column are not significantly different at 0.05 LSD.
