# Supplementary material for: Impact of Sporisorium scitamineum infection on the qualitative traits of commercial cultivars and advanced lines of sugarcane
Source: PLoS One. 2022 May 23;17(5):e0268781. doi: 10.1371/journal.pone.0268781 (PMC9126389; doi:10.1371/journal.pone.0268781)
Supplement: S3 Table — (DOCX) [file pone.0268781.s003.docx]

**Table S3. Effects of whip smut *Sporisorium scitamineum* on fiber percentage of sugarcane cultivars in field screening trial with artificial inoculation.**

| **S. No** | **Varieties** | **Smut**  **Rating** | **Fiber (%)** | | **Increase**  **Percent** | **T value** |
| --- | --- | --- | --- | --- | --- | --- |
|  |  |  | **Inoculated** | **Natural Infection** |  |  |
| 1 | AP-04-68/01 | 0 | 14.49±0.01^b-p^ | 14.50±0.01^b^ | -0.01 | -0.15 |
| 2 | AP-97-56/02 | 0 | 14.65±0.02^a-h^ | 14.62±0.04^a^ | 0.19 | 0.84 |
| 3 | AP-97-69/01 | 0 | 14.26±0.02^h-t^ | 14.25±0.02^e-g^ | 0.07 | 0.75 |
| 4 | AP-98-103/01 | 0 | 14.15±0.02^l-v^ | 14.12±0.02^h-k^ | 0.22 | 2.01 |
| 5 | AP-98-156/02 | 0 | 14.50±0.09^b-o^ | 14.48±0.01^bc^ | 0.16 | 0.29 |
| 6 | AP-98-156/03 | 0 | 14.15±0.03^l-v^ | 14.16±0.01^f-j^ | -0.02 | -0.10 |
| 7 | AP-98-156/04 | 0 | 13.75±0.08^v-z,A-D^ | 13.76±0.03^no^ | -0.11 | -0.28 |
| 8 | AP-98-156/07 | 0 | 14.26±0.02^h-t^ | 14.29±0.05^de^ | -0.22 | -1.10 |
| 9 | AP-97-56/03 | 0 | 14.17±0.01^k-v^ | 14.18±0.01^e-h^ | -0.08 | -0.95 |
| 10 | BPTh-807 | 0 | 12.30±0.04^U^ | 12.34±0.03^OP^ | -0.26 | -1.39 |
| 11 | BP-TJ-651/18 | 0 | 14.38±0.02^f-r^ | 14.37±0.02^cd^ | 0.05 | 0.44 |
| 12 | BP-TJ-651/20 | 0 | 12.48±0.09^Q-U^ | 12.36±0.02^OP^ | 0.90 | 1.16 |
| 13 | CB-2919 | 0 | 14.51±0.03^b-n^ | 14.54±0.02^ab^ | -0.22 | -1.31 |
| 14 | CP-70-530 | 0 | 12.58±0.04^O-U^ | 12.54±0.03^G-J^ | 0.32 | 0.70 |
| 15 | HoTh-318 | 0 | 12.34±0.03^TU^ | 12.33±0.01^OP^ | 0.08 | 0.34 |
| 16 | HoTh-4140 | 0 | 12.43±0.05^R-U^ | 12.35±0.03^OP^ | 0.62 | 1.12 |
| 17 | HoTh-438 | 0 | 12.68±0.05^O-U^ | 12.71±0.04^yz,A-D^ | -0.24 | -1.03 |
| 18 | HoTh-516 | 0 | 12.35±0.04^S-U^ | 12.36±0.03^OP^ | -0.07 | -0.19 |
| 19 | HoTh-544 | 0 | 14.04±0.01^q-x^ | 14.05±0.01^i-l^ | -0.04 | -0.24 |
| 20 | HoTh-610 | 0 | 13.39±0.06^D-I^ | 13.42±0.04^qr^ | -0.29 | -0.72 |
| 21 | QSG-1741 | 0 | 13.45±0.17^C-H^ | 13.32±0.08^rs^ | 0.97 | 0.74 |
| 22 | Roc-16 | 0 | 12.92±0.12^K-P^ | 13.04±0.04^v^ | -0.93 | -1.00 |
| 23 | S-2003-QSSG-776 | 0 | 12.70±0.12^N-U^ | 12.62±0.03^B-H^ | 0.56 | 0.56 |
| 24 | S-2003-US-633 | 0 | 12.42±0.09^R-U^ | 12.32±0.02^OP^ | 0.79 | 1.20 |
| 25 | S-2006-SP-30 | 0 | 12.64±0.05^O-U^ | 12.59±0.09^E-I^ | 0.41 | 0.67 |
| 26 | Th-704 | 0 | 13.12±0.05^H-N^ | 13.10±0.05^uv^ | 0.13 | 0.52 |
| 27 | AP-04-46/03 | 1 | 14.12±0.18^m-w^ | 13.97±0.03^lm^ | 1.04 | 0.71 |
| 28 | HoTh-344 | 1 | 13.43±0.06^C-I^ | 13.40±0.05^qr^ | 0.22 | 0.48 |
| 29 | AP-98-156/06 | 2 | 14.40±0.11^e-r^ | 14.14±0.03^g-j^ | 1.78 | 2.00 |
| 30 | CP-82-2083 | 2 | 14.18±0.14^j-u^ | 14.03±0.02^kl^ | 1.06 | 0.94 |
| 31 | HoTh-518 | 2 | 12.94±0.15^J-P^ | 12.37±0.03^N-P^ | 4.41 | 3.72^*^ |
| 32 | S-2002-HSG-200 | 2 | 14.08±0.10^o-x^ | 13.87±0.03^mn^ | 1.75 | 2.20 |
| 33 | AP-04-59/02 | 3 | 13.68±0.18^x-z,A-F^ | 12.92±0.02^wx^ | 5.53 | 4.16^**^ |
| 34 | AP-04-68/03 | 3 | 12.55±0.10^P-U^ | 12.38±0.00^M-P^ | 1.29 | 1.65 |
| 35 | AP-04-59/03 | 3 | 14.78±0.17^a-g^ | 14.62±0.02^a^ | 1.13 | 0.89 |
| 36 | BPTh-804 | 3 | 13.26±0.21^F-L^ | 12.43±0.03^K-O^ | 6.27 | 4.25^**^ |
| 37 | CPS-1827 | 3 | 13.36±0.19^D-J^ | 12.48±0.01^J-N^ | 6.58 | 4.62^**^ |
| 38 | Chandka | 3 | 13.50±0.17^B-H^ | 12.54±0.03^G-J^ | 7.10 | 5.32^**^ |
| 39 | CO-620 | 3 | 13.83±0.03^u-z,A-C^ | 13.03±0.18^vw^ | 5.79 | 4.21^**^ |
| 40 | CPSG-244-S-2083 | 3 | 12.75±0.16^N-T^ | 12.32±0.03^OP^ | 3.31 | 2.59^*^ |
| 41 | HoTh-419 | 3 | 13.00±0.14^I-O^ | 12.63±0.03^A-G^ | 2.86 | 2.59^*^ |
| 42 | HoTh-424 | 3 | 12.79±0.17^M-R^ | 12.38±0.04^M-P^ | 3.18 | 2.85^*^ |
| 43 | HoTh-513 | 3 | 13.43±0.18^C-H^ | 12.69±0.03^z,A-E^ | 5.46 | 4.09^**^ |
| 44 | HoTh-517 | 3 | 12.69±0.19^O-U^ | 12.37±0.02^N-P^ | 2.50 | 1.69 |
| 45 | S-2003-HOSG-679 | 3 | 12.65±0.17^O-U^ | 12.38±0.02^M-P^ | 2.13 | 1.64 |
| 46 | S-2003-US-160 | 3 | 13.53±0.18^A-H^ | 12.77±0.03^yz^ | 5.60 | 4.21^**^ |
| 47 | Th-720 | 3 | 14.54±0.16^a-m^ | 14.07±0.01^h-l^ | 3.22 | 2.87^*^ |
| 48 | AP-04-68/02 | 4 | 13.19±0.14^G-M^ | 12.65±0.05^A-G^ | 4.11 | 3.81^*^ |
| 49 | B-43405 | 4 | 13.43±0.17^C-H^ | 12.71±0.02^yz,A-D^ | 5.36 | 4.12^**^ |
| 50 | B-46364 | 4 | 13.71±0.22^w-z,A-E^ | 12.73±0.03^yz,AB^ | 7.09 | 4.78^**^ |
| 51 | BP-TJ-15/01 | 4 | 13.38±0.21^D-I^ | 12.35±0.02^OP^ | 7.71 | 5.28^**^ |
| 52 | CPF-229 | 4 | 13.55±0.19^A-G^ | 12.48±0.01^I-M^ | 7.91 | 5.51^**^ |
| 53 | CO-413 | 4 | 14.88±0.15^a-c^ | 14.25±0.02^e-g^ | 4.29 | 3.93^*^ |
| 54 | CP-52-28 | 4 | 14.95±0.16^a^ | 14.44±0.01^bc^ | 3.40 | 3.23^*^ |
| 55 | CP-70-SP-1215 | 4 | 14.60±0.15^a-j^ | 14.15±0.01^f-j^ | 3.04 | 2.98^*^ |
| 56 | CP-85-SP-571 | 4 | 14.41±0.21^d-r^ | 13.17±0.03^tu^ | 8.64 | 6.30^**^ |
| 57 | CSSG-2402 | 4 | 14.01±0.18^r-y^ | 12.58±0.03^F-J^ | 10.20 | 7.09^**^ |
| 58 | CSSG-2476 | 4 | 12.80±0.16^M-R^ | 12.38±0.02^M-P^ | 3.31 | 2.74^*^ |
| 59 | H-86-NSG-311 | 4 | 14.12±0.14^m-w^ | 12.72±0.07^yz,A-C^ | 9.90 | 6.99^**^ |
| 60 | HoTh-316 | 4 | 12.77±0.13^M-S^ | 12.35±0.02^OP^ | 3.25 | 3.09^*^ |
| 61 | HoTh-127 | 4 | 14.77±0.15^a-g^ | 14.26±0.01^d-f^ | 3.40 | 3.23^*^ |
| 62 | HoTh-326 | 4 | 13.37±0.18^D-I^ | 12.61±0.02^C-H^ | 5.66 | 4.07^**^ |
| 63 | HoTh-432 | 4 | 12.71±0.20^N-U^ | 12.42±0.02^L-P^ | 2.31 | 1.45 |
| 64 | HoTh-518 | 4 | 13.35±0.14^D-J^ | 12.67±0.07^z,A-F^ | 5.09 | 4.04^**^ |
| 65 | HoTh-612 | 4 | 12.69±0.17^O-U^ | 12.35±0.03^OP^ | 2.71 | 2.04 |
| 66 | NSG-60 | 4 | 14.62±0.17^a-i^ | 14.16±0.01^f-i^ | 3.12 | 2.73^*^ |
| 67 | Q-88 | 4 | 13.36±0.20^D-J^ | 12.52±0.02^H-L^ | 6.32 | 4.34^**^ |
| 68 | S-2003-CPSG-704 | 4 | 13.93±0.20^t-z,A^ | 12.81±0.03^xy^ | 8.04 | 5.48^**^ |
| 69 | S-2006-SP-18 | 4 | 13.71±0.19^w-z,A-E^ | 12.48±0.01^I-M^ | 8.94 | 6.51^**^ |
| 70 | S-2003-CPSG-193 | 4 | 13.31±0.20^E-K^ | 12.37±0.02^M-P^ | 7.06 | 5.16^**^ |
| 71 | SPSG-3481 | 4 | 14.09±0.20^n-x^ | 12.60±0.01^D-H^ | 10.54 | 7.53^**^ |
| 72 | Th-702 | 4 | 13.81±0.18^u-z,A-C^ | 12.54±0.02^G-K^ | 9.25 | 6.84^**^ |
| 73 | Th-725 | 4 | 14.60±0.16^a-j^ | 14.07±0.01^h-l^ | 3.61 | 3.33^*^ |
| 74 | Th-10 | 4 | 13.61±0.19^yz,A-G^ | 12.38±0.01^M-P^ | 9.06 | 6.67^**^ |
| 75 | AP-04-46/02 | 5 | 14.76±0.14^a-g^ | 14.53±0.01^ab^ | 1.58 | 1.72 |
| 76 | COJ-84 | 5 | 14.84±0.16^a-d^ | 13.55±0.05^p^ | 8.67 | 6.45^**^ |
| 77 | CP-75-1353 | 5 | 14.45±0.03^d-q^ | 13.68±0.17^o^ | 5.29 | 4.08^**^ |
| 78 | HoTh-401 | 5 | 12.65±0.15^O-U^ | 12.41±0.02^L-P^ | 1.90 | 1.71 |
| 79 | HSF-240 | 5 | 14.39±0.18^f-r^ | 13.22±0.04^st^ | 8.09 | 5.58^**^ |
| 80 | NCO-310 | 5 | 14.39±0.17^f-r^ | 13.98±0.02^lm^ | 2.86 | 2.59^*^ |
| 81 | S-2003-US-704 | 5 | 12.87±0.16^L-Q^ | 12.36±0.03^OP^ | 3.93 | 3.11^*^ |
| 82 | S-2006-SP-658 | 5 | 13.57±0.15^z,A-G^ | 13.03±0.04^v^ | 3.95 | 3.21^*^ |
| 83 | AP-98-156/05 | 6 | 14.61±0.20^a-i^ | 14.47±0.01^bc^ | 0.97 | 0.73 |
| 84 | AP-04-59/01 | 6 | 13.47±0.18^C-H^ | 12.38±0.03^M-P^ | 8.06 | 5.56^**^ |
| 85 | AP-98-156/08 | 6 | 14.82±0.09^a-e^ | 14.62±0.01^a^ | 1.38 | 2.22 |
| 86 | CO-639 | 6 | 14.92±0.21^ab^ | 14.28±0.01^de^ | 4.26 | 2.89^*^ |
| 87 | S-2003-HOSG-1626 | 6 | 12.75±0.13^N-T^ | 12.39±0.04^M-P^ | 2.85 | 2.54 |
| 88 | YT-236 | 6 | 14.59±0.15^a-k^ | 14.14±0.01^g-j^ | 3.05 | 2.84^*^ |
| 89 | AP-98-156/01 | 7 | 14.08±0.22^p-x^ | 12.71±0.02^yz,A-C^ | 9.67 | 6.10^**^ |
| 90 | CO-1148 | 7 | 13.41±0.19^C-I^ | 12.64±0.03^A-G^ | 5.69 | 4.07^**^ |
| 91 | COJ-81 | 7 | 14.49±0.11^C-P^ | 13.79±0.09^no^ | 4.79 | 3.84^*^ |
| 92 | CP-59-1059 | 7 | 13.71±0.20^w-z,A-E^ | 12.31±0.02 ^P^ | 10.20 | 7.11^**^ |
| 93 | CP-69-1059 | 7 | 14.79±0.14^a-f^ | 14.05±0.09^j-l^ | 5.06 | 3.96^*^ |
| 94 | HoTh-408 | 7 | 14.16±0.21^k-v^ | 12.36±0.03^OP^ | 12.72 | 8.45^**^ |
| 95 | HoTh-409 | 7 | 14.42±0.22^d-r^ | 13.44±0.06^pq^ | 6.74 | 4.91^**^ |
| 96 | Larkana-2001 | 7 | 13.92±0.21^t-z,AB^ | 12.74±0.03^yz,A^ | 8.49 | 6.38^**^ |
| 97 | S-2002-SFSD-1307 | 7 | 14.14±0.20^m-v^ | 12.89±0.02^x^ | 8.84 | 6.45^**^ |
| 98 | S-2003-HOSG-701 | 7 | 13.71±0.19^w-z,A-E^ | 12.37±0.03^N-P^ | 9.78 | 6.83^**^ |
| 99 | CO-208 | 8 | 13.95±0.23^s-z,A^ | 12.35±0.03^OP^ | 11.47 | 7.65^**^ |
| 100 | CPD-01-359 | 8 | 14.00±0.21^r-z^ | 12.36±0.02^OP^ | 11.71 | 7.83^**^ |
| 101 | Tritan | 8 | 14.26±0.22^h-t^ | 12.58±0.03^F-J^ | 11.81 | 7.93^**^ |
| 102 | CP-29-120 | 9 | 14.57±0.22^a-l^ | 12.81±0.02^xy^ | 12.08 | 8.03^**^ |
| 103 | CSSG-1741 | 9 | 14.36±0.25^g-s^ | 12.73±0.06^yz,AB^ | 11.40 | 7.38^**^ |
| 104 | HoTh-550 | 9 | 14.20±0.21^i-u^ | 12.32±0.03^OP^ | 13.23 | 8.60^**^ |
|  | F-Statistics at df = 103 | | 24.15 | 384.00 |  |  |
|  | LSD 0.05 | | 0.4257 | 0.1133 |  |  |

ns= Non-significant at 0.05, * = significant at 0.05; and ** = highly significant at 0.01 level

Means followed by same letter(s) in the same column are not significantly different at 0.05 LSD
