# Supplementary material for: Impact of Sporisorium scitamineum infection on the qualitative traits of commercial cultivars and advanced lines of sugarcane
Source: PLoS One. 2022 May 23;17(5):e0268781. doi: 10.1371/journal.pone.0268781 (PMC9126389; doi:10.1371/journal.pone.0268781)
Supplement: S4 Table — (DOCX) [file pone.0268781.s004.docx]

**Table S4. Effects of whip smut *Sporisorium scitamineum* on purity percentage of sugarcane cultivars in field screening trial with artificial inoculation.**

| **S. No** | **Varieties** | **Smut**  **Rating** | **Purity (%)** | | **Reduction**  **Percent** | **T value** |
| --- | --- | --- | --- | --- | --- | --- |
|  |  |  | **Inoculated** | **Natural Infection** |  |  |
| 1 | AP-04-68/01 | 0 | 81.86±0.70^D-P^ | 81.87±0.95^H-J^ | 0.01 | -0.01 |
| 2 | AP-97-56/02 | 0 | 81.42±0.20^I-R^ | 81.50±0.09^JK^ | 0.10 | -0.51 |
| 3 | AP-97-69/01 | 0 | 82.92±0.63^t-z,A-D^ | 82.27±0.51^F-I^ | -0.78 | 1.02 |
| 4 | AP-98-103/01 | 0 | 83.29±0.16^o-y^ | 83.25±0.12^w-z,A-D^ | -0.05 | 0.46 |
| 5 | AP-98-156/02 | 0 | 83.12±0.81^r-z,AB^ | 83.13±0.67^x-z,A-E^ | 0.01 | -0.01 |
| 6 | AP-98-156/03 | 0 | 83.46±0.29^n-x^ | 83.42±0.13^s-z,AB^ | -0.04 | 0.10 |
| 7 | AP-98-156/04 | 0 | 84.88±0.25^c-j^ | 84.94±0.08^d-i^ | 0.07 | -0.32 |
| 8 | AP-98-156/07 | 0 | 82.91±0.42^t-z,A-D^ | 82.84±0.65^z,A-F^ | -0.09 | 0.15 |
| 9 | AP-97-56/03 | 0 | 83.59±0.11^m-w^ | 83.70±0.07^p-x^ | 0.14 | -2.00 |
| 10 | BPTh-807 | 0 | 85.96±0.19^a-c^ | 86.17±0.13^ab^ | 0.24 | -2.20 |
| 11 | BP-TJ-651/18 | 0 | 82.74±0.20^v-z,A-F^ | 82.75±0.18^B-G^ | 0.02 | -0.09 |
| 12 | BP-TJ-651/20 | 0 | 86.33±0.29^a^ | 86.28±0.11^a^ | -0.05 | 0.15 |
| 13 | CB-2919 | 0 | 81.55±0.21^H-R^ | 81.57±0.76^JK^ | 0.02 | -0.02 |
| 14 | CP-70-530 | 0 | 84.36±0.17^e-o^ | 84.79±0.65^e-j^ | 0.51 | -0.77 |
| 15 | HoTh-318 | 0 | 86.21±0.18^ab^ | 86.13±0.06^ab^ | -0.09 | 0.51 |
| 16 | HoTh-4140 | 0 | 84.55±0.52^d-n^ | 85.28±0.29^de^ | 0.86 | -1.05 |
| 17 | HoTh-438 | 0 | 84.35±0.23^e-o^ | 84.39±0.22^i-p^ | 0.05 | -0.36 |
| 18 | HoTh-516 | 0 | 86.20±0.19^ab^ | 86.34±0.19^a^ | 0.16 | -0.62 |
| 19 | HoTh-544 | 0 | 82.10±0.13^A-N^ | 82.13±0.21^G-J^ | 0.03 | -0.07 |
| 20 | HoTh-610 | 0 | 83.91±0.15^i-u^ | 83.98±0.10^l-v^ | 0.08 | -0.34 |
| 21 | QSG-1741 | 0 | 83.64±0.11^l-w^ | 83.73±0.21^o-x^ | 0.10 | -0.40 |
| 22 | Roc-16 | 0 | 84.40±0.38^e-n^ | 84.40±0.57^h-o^ | 0.01 | -0.01 |
| 23 | S-2003-QSSG-776 | 0 | 84.14±0.28^f-r^ | 83.96±0.14^l-v^ | -0.22 | 1.17 |
| 24 | S-2003-US-633 | 0 | 85.98±0.18^a-c^ | 86.09±0.11^a-c^ | 0.12 | -0.70 |
| 25 | S-2006-SP-30 | 0 | 84.11±0.23^f-r^ | 84.18±0.20^j-q^ | 0.09 | -0.38 |
| 26 | Th-704 | 0 | 84.14±0.49^f-r^ | 84.09±0.52^k-s^ | -0.05 | 0.15 |
| 27 | AP-04-46/03 | 1 | 83.64±0.45^l-w^ | 84.39±0.69^i-o^ | 0.89 | -1.21 |
| 28 | HoTh-344 | 1 | 83.91±0.42^i-u^ | 84.16±0.43^j-q^ | 0.31 | -1.72 |
| 29 | AP-98-156/06 | 2 | 82.41±0.41^x-z,A-I^ | 83.22±0.35^w-z,A-D^ | 0.97 | -2.67^*^ |
| 30 | CP-82-2083 | 2 | 82.66±0.26^v-z,A-G^ | 83.35±0.25^u-z,A-D^ | 0.83 | -1.35 |
| 31 | HoTh-518 | 2 | 84.70±0.51^d-l^ | 86.35± 0.17^a^ | 1.91 | -3.33^*^ |
| 32 | S-2002-HSG-200 | 2 | 84.25±0.37^e-q^ | 85.28±0.12^de^ | 1.21 | -2.77^*^ |
| 33 | AP-04-59/02 | 3 | 84.58±0.26^d-m^ | 85.52±0.10^b-d^ | 1.10 | -3.40^*^ |
| 34 | AP-04-68/03 | 3 | 85.62±0.27^a-d^ | 86.45±0.02^a^ | 0.96 | -2.90^*^ |
| 35 | AP-04-59/03 | 3 | 80.11±0.28**^U-X^** | 81.12±0.32^K^ | 1.24 | -3.19^*^ |
| 36 | BPTh-804 | 3 | 84.93±0.37^c-j^ | 86.09±0.20^a-c^ | 1.34 | -3.46^*^ |
| 37 | CPS-1827 | 3 | 83.75±0.41^k-v^ | 85.09±0.12^d-h^ | 1.57 | -3.51^*^ |
| 38 | Chandka | 3 | 82.99±0.40^s-z,A-C^ | 83.61±0.14^q-x^ | 0.74 | -1.92 |
| 39 | CO-620 | 3 | 83.93±0.42^h-t^ | 85.21±0.15^d-f^ | 1.50 | -3.47^*^ |
| 40 | CPSG-244-S-2083 | 3 | 85.02±0.28^c-h^ | 86.15±0.14^ab^ | 1.31 | -3.18^*^ |
| 41 | HoTh-419 | 3 | 82.87±0.28^t-z,A-E^ | 84.00±0.17^l-u^ | 1.35 | -3.23^*^ |
| 42 | HoTh-424 | 3 | 85.19±0.30^b-f^ | 86.43±0.20^a^ | 1.42 | -3.19^*^ |
| 43 | HoTh-513 | 3 | 83.17±0.41^q-z,A^ | 84.32±0.16^i-p^ | 1.38 | -3.31^*^ |
| 44 | HoTh-517 | 3 | 84.99±0.42^c-i^ | 86.38±0.11^a^ | 1.61 | -3.91^*^ |
| 45 | S-2003-HOSG-679 | 3 | 85.33±0.36^a-e^ | 86.45±0.09^a^ | 1.30 | -3.58^*^ |
| 46 | S-2003-US-160 | 3 | 83.59±0.39^m-w^ | 84.76±0.16^e-k^ | 1.37 | -3.85^*^ |
| 47 | Th-720 | 3 | 81.16±0.43^K-U^ | 82.39±0.18^F-H^ | 1.49 | -3.08^*^ |
| 48 | AP-04-68/02 | 4 | 82.33±0.35^yz,A-J^ | 84.08±0.14^k-t^ | 2.09 | -4.16^**^ |
| 49 | B-43405 | 4 | 82.92±0.32^t-z,A-D^ | 84.43±0.11^h-n^ | 1.79 | -3.73^*^ |
| 50 | B-46364 | 4 | 83.22±0.38^p-z^ | 84.53±0.15^f-l^ | 1.54 | -3.71^*^ |
| 51 | BP-TJ-15/01 | 4 | 84.45±0.44^e-n^ | 86.28±0.07^a^ | 2.13 | -4.29^**^ |
| 52 | CPF-229 | 4 | 82.70±0.41^v-z,A-F^ | 84.49±0.12^g-m^ | 2.13 | -4.28^**^ |
| 53 | CO-413 | 4 | 80.74±0.54**^Q-W^** | 82.85±0.24^yz,A-F^ | 2.54 | -4.64^**^ |
| 54 | CP-52-28 | 4 | 81.80±0.49^E-R^ | 83.52±0.14^q-z^ | 2.06 | -4.07^**^ |
| 55 | CP-70-SP-1215 | 4 | 82.13±0.44^z,A-M^ | 83.46±0.11^r-z,A^ | 1.59 | -3.31^*^ |
| 56 | CP-85-SP-571 | 4 | 81.94±0.34^C-0^ | 83.52±0.15^q-z^ | 1.88 | -3.46^*^ |
| 57 | CSSG-2402 | 4 | 81.58±0.44^G-R^ | 83.72±0.15^o-x^ | 2.56 | -4.93^**^ |
| 58 | CSSG-2476 | 4 | 84.81±0.42^d-k^ | 86.44±0.09^a^ | 1.89 | -3.47^*^ |
| 59 | H-86-NSG-311 | 4 | 82.80±0.43^u-z,A-E^ | 84.48±0.06 ^h-m^ | 1.98 | -4.02^*^ |
| 60 | HoTh-316 | 4 | 85.04±0.32^c-g^ | 86.31±0.12^a^ | 1.47 | -3.27^*^ |
| 61 | HoTh-127 | 4 | 81.35±0.40^I-S^ | 82.73±0.12^C-G^ | 1.67 | -3.66^*^ |
| 62 | HoTh-326 | 4 | 82.61±0.37^w-z,A-H^ | 83.90±0.10^l-w^ | 1.55 | -3.51^*^ |
| 63 | HoTh-432 | 4 | 84.76±0.47^d-k^ | 86.63±0.11^a^ | 2.16 | -4.67^**^ |
| 64 | HoTh-518 | 4 | 83.60±0.29^l-w^ | 84.17±0.35^j-q^ | 0.67 | -2.18 |
| 65 | HoTh-612 | 4 | 84.93±0.33^c-j^ | 86.26±0.17^a^ | 1.53 | -3.52^*^ |
| 66 | NSG-60 | 4 | 81.68±0.44^F-R^ | 83.53±0.07^q-y^ | 2.21 | -4.37^**^ |
| 67 | Q-88 | 4 | 81.92±0.43^C-O^ | 83.40±0.08^t-z,A-C^ | 1.77 | -3.63^*^ |
| 68 | S-2003-CPSG-704 | 4 | 83.22±0.45^p-z^ | 84.96±0.13^d-i^ | 2.05 | -4.04^**^ |
| 69 | S-2006-SP-18 | 4 | 81.44±0.43^I-R^ | 83.27±0.03^w-z,A-D^ | 2.19 | -4.46^**^ |
| 70 | S-2003-CPSG-193 | 4 | 84.78±0.47^d-k^ | 86.42±0.09^a^ | 1.90 | -3.70^*^ |
| 71 | SPSG-3481 | 4 | 82.20±0.39^yz,A-L^ | 83.83±0.06 ^m-w^ | 1.95 | -3.71^*^ |
| 72 | Th-702 | 4 | 82.72±0.39^v-z,A-F^ | 84.12±0.13^j-r^ | 1.67 | -3.77^*^ |
| 73 | Th-725 | 4 | 81.03±0.46^N-V^ | 82.46 ±0.17^E-H^ | 1.74 | -3.82^*^ |
| 74 | Th-10 | 4 | 84.29±0.45^e-p^ | 86.37±0.06^a^ | 2.42 | -4.59^**^ |
| 75 | AP-04-46/02 | 5 | 77.58±0.39**^Z^** | 79.89±0.10^L^ | 2.90 | -5.26^**^ |
| 76 | COJ-84 | 5 | 80.23±0.44**^t-x^** | 82.70±0.26^D-G^ | 2.99 | -5.43^**^ |
| 77 | CP-75-1353 | 5 | 82.68±0.48^v-z,A-G^ | 85.17±0.06^d-g^ | 2.92 | -5.28^**^ |
| 78 | HoTh-401 | 5 | 84.08±0.41^g-s^ | 86.63±0.09^a^ | 2.94 | -5.39^**^ |
| 79 | HSF-240 | 5 | 81.06±0.46^M-U^ | 83.42±0.11^s-z,AB^ | 2.83 | -5.00^**^ |
| 80 | NCO-310 | 5 | 81.79±0.33^E-R^ | 84.34±0.17^i-p^ | 3.02 | -5.46^**^ |
| 81 | S-2003-US-704 | 5 | 83.54±0.46^m-w^ | 86.34±0.17^a^ | 3.24 | -5.50^**^ |
| 82 | S-2006-SP-658 | 5 | 82.24±0.47^yz,A-K^ | 84.40±0.12^h-o^ | 2.55 | -4.89^**^ |
| 83 | AP-98-156/05 | 6 | 81.10±0.50^L-U^ | 83.76±0.10^n-x^ | 3.17 | -5.83^**^ |
| 84 | AP-04-59/01 | 6 | 83.46±0.53^n-x^ | 86.40±0.15^a^ | 3.40 | -5.93^**^ |
| 85 | AP-98-156/08 | 6 | 78.73±0.50**^Y^** | 80.99±0.09^K^ | 2.79 | -4.91^**^ |
| 86 | CO-639 | 6 | 78.75±0.53**^Y^** | 81.64±0.11^I-K^ | 3.54 | -5.99^**^ |
| 87 | S-2003-HOSG-1626 | 6 | 83.87±0.56^j-u^ | 86.50±0.22^a^ | 3.04 | -5.59^**^ |
| 88 | YT-236 | 6 | 80.72±0.45^R-W^ | 83.29±0.18^v-z,A-D^ | 3.08 | -5.74^**^ |
| 89 | AP-98-156/01 | 7 | 81.27±0.53^J-T^ | 84.40±0.08^i-o^ | 3.71 | -6.23^**^ |
| 90 | CO-1148 | 7 | 80.92±0.50^O-W^ | 84.07±0.16^l-t^ | 3.74 | -6.26^**^ |
| 91 | COJ-81 | 7 | 79.87±0.52**^WX^** | 82.82±0.22^A-F^ | 3.57 | -5.72^**^ |
| 92 | CP-59-1059 | 7 | 82.33±0.51^yz,A-J^ | 86.07±0.11^a-c^ | 4.35 | -6.62^**^ |
| 93 | CP-69-1059 | 7 | 79.24±0.45**^XY^** | 82.32±0.08^F-I^ | 3.74 | -6.25^**^ |
| 94 | HoTh-408 | 7 | 82.20±0.55^yz,A-L^ | 86.34±0.17^a^ | 4.79 | -6.98^**^ |
| 95 | HoTh-409 | 7 | 80.75±0.34^P-W^ | 84.09±0.20^k-t^ | 3.96 | -6.68^**^ |
| 96 | Larkana-2001 | 7 | 81.23±0.40^J-T^ | 84.56±0.16^f-l^ | 3.93 | -6.64^**^ |
| 97 | S-2002-SFSD-1307 | 7 | 82.05±0.43^B-N^ | 85.41±0.11^c-e^ | 3.94 | -6.65^**^ |
| 98 | S-2003-HOSG-701 | 7 | 82.34±0.50^yz,A-J^ | 86.35±0.14^a^ | 4.65 | -7.74^**^ |
| 99 | CO-208 | 8 | 81.83±0.40^D-Q^ | 86.24±0.16^a^ | 5.10 | -8.25^**^ |
| 100 | CPD-01-359 | 8 | 82.01±0.53^C-O^ | 86.30±0.10^a^ | 4.98 | -8.03^**^ |
| 101 | Tritan | 8 | 79.48±0.52**^XY^** | 83.72±0.15^o-x^ | 5.06 | -8.12^**^ |
| 102 | CP-29-120 | 9 | 76.11±0.57**^a^** | 84.97±0.12^d-i^ | 10.42 | -13.16^**^ |
| 103 | CSSG-1741 | 9 | 79.94±0.52**^V-X^** | 84.52±0.07^g-m^ | 5.42 | -8.39^**^ |
| 104 | HoTh-550 | 9 | 80.29±0.48^S-X^ | 86.12±0.14^ab^ | 6.77 | -10.17^**^ |
|  | F-Statistics at df = 103 | | 23.14 | 38.56 |  |  |
|  | LSD 0.05 | | 1.1041 | 0.6887 |  |  |

ns= Non-significant at 0.05, * = significant at 0.05; and ** = highly significant at 0.01 level

Means followed by same letter(s) in the same column are not significantly different at 0.05 LSD
