# Supplementary material for: Impact of Sporisorium scitamineum infection on the qualitative traits of commercial cultivars and advanced lines of sugarcane
Source: PLoS One. 2022 May 23;17(5):e0268781. doi: 10.1371/journal.pone.0268781 (PMC9126389; doi:10.1371/journal.pone.0268781)
Supplement: S5 Table — (DOCX) [file pone.0268781.s005.docx]

**Table S5. Effects of whip smut *Sporisorium scitamineum* on CCS percentage of sugarcane varieties/cultivars in field screening trial with artificial inoculation.**

| **S. No** | **Varieties** | **Smut**  **Rating** | **CCS (%)** | | **Reduction**  **Percent** | **T value** |
| --- | --- | --- | --- | --- | --- | --- |
|  |  |  | **Inoculated** | **Natural Infection** |  |  |
| 1 | AP-04-68/01 | 0 | 11.11±0.10^D-G^ | 11.12±0.14^O^ | 0.13 | -0.06 |
| 2 | AP-97-56/02 | 0 | 10.43±0.01^J^ | 10.44±0.02^Q^ | 0.12 | -0.39 |
| 3 | AP-97-69/01 | 0 | 11.91±0.07^x-z,A^ | 11.97±0.07^J^ | 0.47 | -0.60 |
| 4 | AP-98-103/01 | 0 | 12.09±0.03^v-z^ | 12.12±0.03^F-J^ | 0.21 | -0.93 |
| 5 | AP-98-156/02 | 0 | 11.20±0.15^D-F^ | 11.24±0.18^NO^ | 0.40 | -0.24 |
| 6 | AP-98-156/03 | 0 | 12.13±0.02^t-y^ | 12.15±0.02^F-H^ | 0.14 | -0.74 |
| 7 | AP-98-156/04 | 0 | 13.17±0.04^d-i^ | 13.22±0.05^z^ | 0.36 | -0.92 |
| 8 | AP-98-156/07 | 0 | 11.68±0.12^A-C^ | 11.73±0.17^K^ | 0.44 | -0.39 |
| 9 | AP-97-56/03 | 0 | 12.27±0.05^s-x^ | 12.26±0.05^FG^ | -0.03 | 0.20 |
| 10 | BPTh-807 | 0 | 13.89±0.04^ab^ | 13.94±0.05^d-h^ | 0.38 | -0.74 |
| 11 | BP-TJ-651/18 | 0 | 11.39±0.01^B-D^ | 11.40± 0.02^MN^ | 0.09 | -0.32 |
| 12 | BP-TJ-651/20 | 0 | 12.94±0.04^g-m^ | 12.88±0.03^B^ | -0.46 | 1.54 |
| 13 | CB-2919 | 0 | 10.79±0.07^g-j^ | 10.72±0.22^P^ | -0.65 | 0.28 |
| 14 | CP-70-530 | 0 | 14.12±0.07^a^ | 14.09±0.07^b-d^ | -0.21 | 0.30 |
| 15 | HoTh-318 | 0 | 13.19±0.02^d-i^ | 13.21±0.02^z,A^ | 0.14 | -0.70 |
| 16 | HoTh-4140 | 0 | 13.83±0.01^ab^ | 13.81±0.03^h-n^ | -0.17 | 0.90 |
| 17 | HoTh-438 | 0 | 13.26±0.04^d-h^ | 13.24±0.02^z^ | -0.21 | 0.48 |
| 18 | HoTh-516 | 0 | 13.88±0.03^ab^ | 13.91±0.02^e-i^ | 0.22 | -0.47 |
| 19 | HoTh-544 | 0 | 12.10±0.01^v-z^ | 12.09±0.01^H-J^ | -0.10 | 0.83 |
| 20 | HoTh-610 | 0 | 13.16±0.05^d-j^ | 13.17±0.02^z,A^ | 0.05 | -0.12 |
| 21 | QSG-1741 | 0 | 13.17±0.04^d-i^ | 13.18±0.02^z,A^ | 0.11 | -0.47 |
| 22 | Roc-16 | 0 | 13.74±0.0^bc^ | 13.75±0.06^j-p^ | 0.02 | -0.06 |
| 23 | S-2003-QSSG-776 | 0 | 13.87±0.04^ab^ | 13.90±0.02^f-j^ | 0.23 | -0.73 |
| 24 | S-2003-US-633 | 0 | 14.11±0.04^ab^ | 14.08±0.05^b-d^ | -0.26 | 0.87 |
| 25 | S-2006-SP-30 | 0 | 13.94±0.04^ab^ | 13.93±0.02^d-h^ | -0.07 | 0.19 |
| 26 | Th-704 | 0 | 13.43±0.16^cd^ | 13.43±0.17^u-x^ | -0.01 | 0.00 |
| 27 | AP-04-46/03 | 1 | 12.08±0.06^v-z^ | 12.22±0.01^F-H^ | 1.17 | -2.64^*^ |
| 28 | HoTh-344 | 1 | 13.37±0.27^de^ | 13.42±0.20^v-y^ | 0.41 | -0.16 |
| 29 | AP-98-156/06 | 2 | 11.28±0.13^DE^ | 11.57±0.03^KL^ | 2.58 | -2.89^*^ |
| 30 | CP-82-2083 | 2 | 11.83±0.09^yz,A^ | 12.43±0.05^DE^ | 4.83 | -4.29^**^ |
| 31 | HoTh-518 | 2 | 12.88±0.15^i-n^ | 13.59±0.03^p-u^ | 5.22 | -4.58^**^ |
| 32 | S-2002-HSG-200 | 2 | 12.43± 0.15^p-v^ | 13.32±0.04^w-z^ | 6.67 | -5.41^**^ |
| 33 | AP-04-59/02 | 3 | 13.06±0.17^d-k^ | 13.57±0.03^r-v^ | 3.76 | -3.41^*^ |
| 34 | AP-04-68/03 | 3 | 13.37±0.16^c-e^ | 13.82±0.03^g-m^ | 3.21 | -3.06^*^ |
| 35 | AP-04-59/03 | 3 | 10.02±0.12 ^K^ | 10.43±0.04^Q^ | 3.94 | -3.69^*^ |
| 36 | BPTh-804 | 3 | 11.82±0.12^yz,A^ | 12.28±0.06^EF^ | 3.75 | -3.36^*^ |
| 37 | CPS-1827 | 3 | 13.14±0.14^d-j^ | 14.01±0.06^c-f^ | 6.20 | -5.29^**^ |
| 38 | Chandka | 3 | 12.84±0.15^i-o^ | 13.89±0.02^f-k^ | 7.53 | -6.57^**^ |
| 39 | CO-620 | 3 | 12.79±0.16^j-p^ | 13.53±0.03^r-v^ | 5.45 | -4.84^**^ |
| 40 | CPSG-244-S-2083 | 3 | 14.05±0.11^ab^ | 14.44±0.06^a^ | 2.68 | -2.98^*^ |
| 41 | HoTh-419 | 3 | 13.17±0.14^d-i^ | 13.81±0.01^g-m^ | 4.64 | -4.50^**^ |
| 42 | HoTh-424 | 3 | 11.84±0.17^yz,A^ | 12.45±0.07^D^ | 4.89 | -4.88^**^ |
| 43 | HoTh-513 | 3 | 13.00±0.13^e-l^ | 13.73±0.03^k-q^ | 5.31 | -4.87^**^ |
| 44 | HoTh-517 | 3 | 13.97±0.12^ab^ | 14.44±0.06^a^ | 3.26 | -3.14^*^ |
| 45 | S-2003-HOSG-679 | 3 | 13.30±0.11^d-g^ | 13.93±0.07^d-h^ | 4.52 | -4.09^**^ |
| 46 | S-2003-US-160 | 3 | 12.97±0.14^f-l^ | 13.75±0.03^i-p^ | 5.66 | -5.28^**^ |
| 47 | Th-720 | 3 | 10.64±0.12^H-J^ | 11.17±0.04^O^ | 4.73 | -4.16^**^ |
| 48 | AP-04-68/02 | 4 | 12.85±0.15^i-o^ | 13.62±0.04^o-t^ | 5.65 | -5.25^**^ |
| 49 | B-43405 | 4 | 12.86±0.18^i-o^ | 13.56±0.04^r-v^ | 5.17 | -4.72^**^ |
| 50 | B-46364 | 4 | 12.53± 0.11^n-s^ | 13.22±0.07^z^ | 5.25 | -4.80^**^ |
| 51 | BP-TJ-15/01 | 4 | 12.85±0.14^i-o^ | 13.59±0.02^p-u^ | 5.42 | -4.91^**^ |
| 52 | CPF-229 | 4 | 12.74±0.18^k-q^ | 13.97±0.04^c-g^ | 9.02 | -8.43^**^ |
| 53 | CO-413 | 4 | 9.97±0.16^KL^ | 10.72±0.06^P^ | 7.04 | -5.97^**^ |
| 54 | CP-52-28 | 4 | 9.78±0.11^K-M^ | 10.70±0.04^P^ | 8.56 | -6.83^**^ |
| 55 | CP-70-SP-1215 | 4 | 10.71±0.13^H-J^ | 11.20±0.03^O^ | 4.40 | -4.11^**^ |
| 56 | CP-85-SP-571 | 4 | 11.15±0.16^D-G^ | 13.20±0.03^z,A^ | 15.53 | -12.32^**^ |
| 57 | CSSG-2402 | 4 | 11.79±0.17^yz,A^ | 13.91±0.02^e-i^ | 15.23 | -12.09^**^ |
| 58 | CSSG-2476 | 4 | 12.85±0.15^i-o^ | 13.58±0.02^q-v^ | 5.35 | -4.94^**^ |
| 59 | H-86-NSG-311 | 4 | 12.08±0.17^v-z^ | 13.90±0.02^f-j^ | 13.13 | -10.32^**^ |
| 60 | HoTh-316 | 4 | 11.83±0.09^yz,A^ | 12.43±0.07^DE^ | 4.83 | -4.18^**^ |
| 61 | HoTh-127 | 4 | 10.66±0.13^H-J^ | 11.19±0.03^O^ | 4.78 | -4.14^**^ |
| 62 | HoTh-326 | 4 | 12.83±0.13^i-o^ | 13.27±0.04^yz^ | 3.26 | -3.17^*^ |
| 63 | HoTh-432 | 4 | 12.51±0.11^n-s^ | 13.28±0.05^x-z^ | 5.78 | -5.22^**^ |
| 64 | HoTh-518 | 4 | 13.11±0.15^d-j^ | 13.53±0.05^s-v^ | 3.10 | -2.99^*^ |
| 65 | HoTh-612 | 4 | 13.26±0.14^d-h^ | 13.82±0.02^g-l^ | 4.06 | -4.03^*^ |
| 66 | NSG-60 | 4 | 10.56±0.12^IJ^ | 11.54±0.07^LM^ | 8.54 | -6.61^**^ |
| 67 | Q-88 | 4 | 12.66± 0.17^l-r^ | 13.88±0.02^f-k^ | 8.80 | -6.90^**^ |
| 68 | S-2003-CPSG-704 | 4 | 12.50±0.16^o-t^ | 13.89±0.03^f-k^ | 10.00 | -9.10^**^ |
| 69 | S-2006-SP-18 | 4 | 12.27±0.13^s-x^ | 13.87±0.03^f-k^ | 11.57 | -10.22^**^ |
| 70 | S-2003-CPSG-193 | 4 | 13.33±0.14^d-f^ | 14.07±0.05^b-e^ | 5.28 | -4.84^**^ |
| 71 | SPSG-3481 | 4 | 12.00±0.16^w-z,A^ | 13.93±0.03^d-h^ | 13.86 | -11.71^**^ |
| 72 | Th-702 | 4 | 12.51±0.12^n-s^ | 13.31±0.05^w-z^ | 6.02 | -5.12^**^ |
| 73 | Th-725 | 4 | 10.44±0.13^J^ | 12.13±0.02^F-I^ | 13.88 | -11.73^**^ |
| 74 | Th-10 | 4 | 12.91±0.16^h-m^ | 13.80±0.02^h-n^ | 6.51 | -5.37^**^ |
| 75 | AP-04-46/02 | 5 | 9.60±0.12^LM^ | 10.47±0.04^Q^ | 8.35 | -6.58^**^ |
| 76 | COJ-84 | 5 | 12.41±0.18^q-v^ | 13.76±0.04^i-o^ | 9.84 | -8.87^**^ |
| 77 | CP-75-1353 | 5 | 11.19±0.17^D-F^ | 12.23±0.08^F-H^ | 8.47 | -7.04^**^ |
| 78 | HoTh-401 | 5 | 13.20±0.20^d-i^ | 14.11±0.04^bc^ | 6.44 | -5.32^**^ |
| 79 | HSF-240 | 5 | 10.96±0.16^E-H^ | 12.43±0.04^DE^ | 11.78 | -10.37^**^ |
| 80 | NCO-310 | 5 | 11.16±0.14^D-G^ | 12.64±0.03^C^ | 11.73 | -10.35^**^ |
| 81 | S-2003-US-704 | 5 | 12.52±0.15^n-s^ | 13.86±0.03^f-k^ | 9.66 | -8.32^**^ |
| 82 | S-2006-SP-658 | 5 | 12.59±0.16^m-s^ | 13.47±0.03^t-w^ | 6.57 | -6.44^**^ |
| 83 | AP-98-156/05 | 6 | 10.46±0.12^J^ | 11.43±0.02^LM^ | 8.53 | -7.17^**^ |
| 84 | AP-04-59/01 | 6 | 12.41±0.15^q-v^ | 13.86±0.03^f-k^ | 10.48 | -9.20^**^ |
| 85 | AP-98-156/08 | 6 | 9.43±0.12 ^MN^ | 10.42±0.02^Q^ | 9.49 | -8.22^**^ |
| 86 | CO-639 | 6 | 9.22±0.13^N^ | 10.50±0.04^Q^ | 12.17 | -10.45^**^ |
| 87 | S-2003-HOSG-1626 | 6 | 12.74±0.20 ^k-q^ | 13.99±0.04^c-f^ | 8.98 | -7.27^**^ |
| 88 | YT-236 | 6 | 10.43±0.15^J^ | 11.97±0.07^IJ^ | 12.83 | -10.53^**^ |
| 89 | AP-98-156/01 | 7 | 11.76±0.17^z,AB^ | 13.50±0.04^s-v^ | 12.93 | -10.67^**^ |
| 90 | CO-1148 | 7 | 12.36±0.17^r-w^ | 13.87±0.03^f-k^ | 10.89 | -9.46^**^ |
| 91 | COJ-81 | 7 | 10.46±0.16^J^ | 12.11±0.03^G-J^ | 13.65 | -11.63^**^ |
| 92 | CP-59-1059 | 7 | 12.49±0.18^o-u^ | 14.01±0.03^c-f^ | 10.82 | -9.40^**^ |
| 93 | CP-69-1059 | 7 | 9.72±0.13^K-M^ | 11.27±0.04^NO^ | 13.80 | -11.69^**^ |
| 94 | HoTh-408 | 7 | 11.79±0.13^yz,A^ | 13.64±0.04^n-s^ | 13.59 | -11.51^**^ |
| 95 | HoTh-409 | 7 | 10.84±0.19^F-I^ | 13.05±0.05^A^ | 16.92 | -14.17^**^ |
| 96 | Larkana-2001 | 7 | 12.02±0.17^w-z,A^ | 13.87±0.04^f-k^ | 13.34 | -11.21^**^ |
| 97 | S-2002-SFSD-1307 | 7 | 11.82±0.16^yz,A^ | 13.67±0.02^l-s^ | 13.53 | -11.41^**^ |
| 98 | S-2003-HOSG-701 | 7 | 12.12±0.14^u-z^ | 13.65±0.04^m-s^ | 11.23 | -9.71^**^ |
| 99 | CO-208 | 8 | 12.00±0.22^w-z,A^ | 14.18±0.05^b^ | 15.39 | -12.22^**^ |
| 100 | CPD-01-359 | 8 | 11.81±0.16^yz,A^ | 13.80±0.03^h-n^ | 14.43 | -11.92^**^ |
| 101 | Tritan | 8 | 11.35±0.14^CD^ | 13.64±0.03^n-s^ | 16.85 | -14.04^**^ |
| 102 | CP-29-120 | 9 | 9.43±0.19^MN^ | 13.89±0.03^f-k^ | 32.10 | -24.25^**^ |
| 103 | CSSG-1741 | 9 | 10.74±0.22^H-J^ | 13.69±00.05^l-r^ | 21.53 | -16.82^**^ |
| 104 | HoTh-550 | 9 | 11.25±0.21^DE^ | 14.01±0.03^c-f^ | 19.71 | -15.18^**^ |
|  | F-Statistics at df = 103 | | 81.27 | 367.64 |  | |
|  | LSD 0.05 | | 0.3722 | 0.1625 |  | |

ns= Non-significant at 0.05, * = significant at 0.05; and ** = highly significant at 0.01 level

Means followed by same letter(s) in the same column are not significantly different at 0.05 LSD
